# Application of Inorganic Nanomaterials in Cultural Heritage Conservation, Risk of Toxicity, and Preventive Measures

**DOI:** 10.3390/nano13091454

**Published:** 2023-04-24

**Authors:** Luz Stella Gomez-Villalba, Ciro Salcines, Rafael Fort

**Affiliations:** 1 Institute of Geosciences, Spanish National Research Council, Complutense University of Madrid (CSIC, UCM), Calle Dr. Severo Ochoa 7, Planta 4, 28040 Madrid, Spain; 2 Infrastructures Service, Health and Safety Unit, University of Cantabria, Pabellón de Gobierno, Avenida de los Castros 54, 39005 Santander, Spain

**Keywords:** cultural heritage conservation, nanomaterials, nanotoxicity, prevention measurements, spraying, brushing, cleaning, personal protection equipment, control banding, international regulations

## Abstract

Nanotechnology has allowed for significant progress in architectural, artistic, archaeological, or museum heritage conservation for repairing and preventing damages produced by deterioration agents (weathering, contaminants, or biological actions). This review analyzes the current treatments using nanomaterials, including consolidants, biocides, hydrophobic protectives, mechanical resistance improvers, flame-retardants, and multifunctional nanocomposites. Unfortunately, nanomaterials can affect human and animal health, altering the environment. Right now, it is a priority to stop to analyze its advantages and disadvantages. Therefore, the aims are to raise awareness about the nanotoxicity risks during handling and the subsequent environmental exposure to all those directly or indirectly involved in conservation processes. It reports the human–body interaction mechanisms and provides guidelines for preventing or controlling its toxicity, mentioning the current toxicity research of main compounds and emphasizing the need to provide more information about morphological, structural, and specific features that ultimately contribute to understanding their toxicity. It provides information about the current documents of international organizations (European Commission, NIOSH, OECD, Countries Normative) about worker protection, isolation, laboratory ventilation control, and debris management. Furthermore, it reports the qualitative risk assessment methods, management strategies, dose control, and focus/receptor relationship, besides the latest trends of using nanomaterials in masks and gas emissions control devices, discussing their risk of toxicity.

## 1. Introduction

Today, nanotechnology has become the primary alternative for progress in fields as diverse as electronics, aeronautics, telecommunications, energy, pharmaceuticals, and biomedicine. Its achievements in the construction and fine art sectors have allowed for progress in new techniques that include nanotechnology to improve the properties of materials and increase their quality.

One of the main problems in different materials, such as those used in construction, ceramics, or fine arts, is to stop the deterioration process, repair, or prevent future wear due to aggressive external agents, which ultimately lead to its destruction. Most of these materials come from samples of cultural heritage interest. Their destruction prevents the preservation of their characteristics, which can have fatal consequences over time, leaving an irreparable historical void [1,2].

With the arrival of nanotechnology in recent decades, the construction [3], museum, archaeology, and arts [4] saw the opportunity to solve this significant problem [1,5,6,7]. It 1was the opportunity to use based nanomaterials with consolidating [1], water repellent [8,9,10], biocidal [11,12], or fire retardant [13] properties [14]. Over time, different research has reported the progress in specific fields of cultural heritage conservation [1,15,16,17,18,19,20].

Despite its success, in recent years, it has been facing a new urgent problem: its toxicity [21]. Over the years of investigation on the risks of nanotoxicity, international organizations have led to creating committees, special bulletins, and databases to report the results of toxicological tests and updated cases about the mortality rates and new diseases linked to nanomaterials’ contact with the human body and, generally, with living beings [22,23,24,25,26]. However, updating reports on the ecotoxicity risk of nanoengineering materials with specific physicochemical properties is essential. Among them, it is urgent to consider that their interaction could lead to critical genetic modifications affecting entire populations in areas close to industrial or mining plants or ecological changes in the soil or water supply, rivers, or wetlands [27,28,29]. These sceneries imply a high risk for the fauna, flora, and, therefore, agriculture or pisciculture products, ultimately affecting the food chain or the water in surface and underground deposits [30,31].

Usually, the operators and personnel involved in conservation activities must be aware of the risks they are exposed to. Nanomaterials are generally handled under the same conditions as any other product. A high potential risk appears when ignoring ventilation, body protection, appropriate handling requirements, and waste management. Brushing, spraying, and immersion are the most common ways to apply nanomaterials in conservation works [32]. In addition, sometimes the surfaces must be polished or ground. Direct contact with nanomaterial emissions might harm the operator and all those who are in the surrounding regions. For instance, during a procedure involving spraying, there is a risk of toxicity due to the ease of inhalation through the nasal way. The air and water transport dispersion would easily affect living beings. In addition, the current release of security regulations for the operators’ protection, the handling of nanomaterials, and derived waste [33,34] do not reach the different specialized groups regarding this subject. In some countries, security during the handling or storage of nanomaterials is not considered a priority, mainly due to gaps in relevant information about standard protocols that guarantee adequate protection measures [35,36,37]. Moreover, the situation is aggravated because the same operators or managers reject protecting themselves, underestimating the hazard [3].

Within the variety of nanoproducts applied in conservation processes, reports indicate toxicity risks, such as those that occur with single or multi-walled carbon nanotubes (SWCNT and MWCMT), silica, zinc oxide, and metals such as silver or hybrid compounds that could be highly toxic if there are not necessary prevention measures [38,39]. As an example, one of the most relevant cases is the use of nano titanium dioxide, with multiple applications in sectors as diverse as cosmetics [40], dentistry [41], food [42], and photovoltaic energy [43], improving its photocatalytic and self-cleaning functions when combined with ZnO [44]. Over the years, the assessment of its toxicity has gone from being an inert nanomaterial for human and ecosystem health to becoming a highly toxic nanomaterial [45]. For instance, as early as 2012, questions began to be raised about whether using sunscreens with nanometric titanium dioxide was safe for health, as they penetrated through the skin [46]. Progress in research on the subject has determined that even a minimal amount does not prevent systemic oxidative stress [47] and that its inhalation can have carcinogenic effects [48]. Moreover, its accumulation due to creams debris also alters beaches where aquatic organisms are highly affected [45,49]. There is a risk of oral ingestion in lip balms [50].

Given that nano titanium oxide is highly toxic, as evidenced by the progress in research, and considering that its use in the heritage conservation sector is quite extensive as a biocide and self-cleaning nanomaterial [1], it is expected that constant exposure is one of the most outstanding examples of why it is necessary to take preventive measures. This protection would also apply to the different potentially harmful nano products.

Due to the importance of this subject, the present review seeks to bring the advances in the field of the conservation of construction materials, ceramics, archaeology, and fine arts, many of them cataloged as cultural heritage using nanomaterials, as well as to identify the possible risks of nanotoxicity, informing about its correct handling throughout all the stages of the process. Additionally, it provides information about the issue’s state regarding nanomaterials’ toxicity and prevention norms or those aspects that require further investigation or clarification.

In this way, it seeks to raise awareness among the personnel involved while handling nanomaterials regarding the risk of exposure to nanomaterials and their effects on the ecosystem, intending that the information provided serves as a basis for current and future generations.

## 2. Deterioration of Cultural Heritage Materials

The main mechanisms directly affecting the historical materials are those produced by environmental [51] or anthropic [52] action. These factors increase the deterioration caused by aggressive agents that destroy architectural, sculptural, archaeological, paleontological, or pictorial materials [53,54,55,56,57].

**Figure 1:**
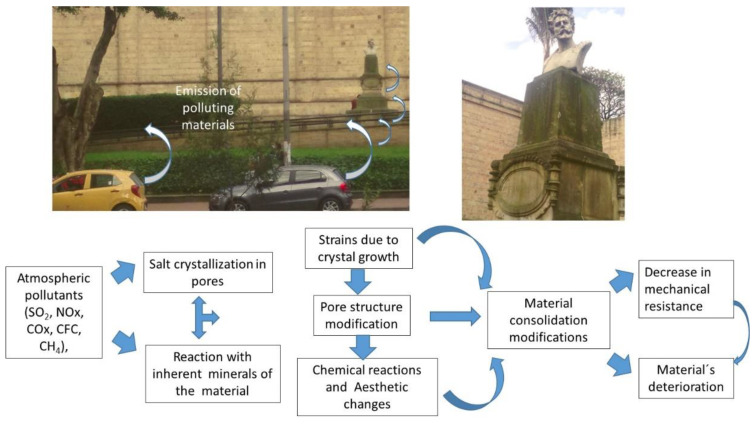
Deterioration of a sculpture and the façade of a museum by urban pollution agents.

Among the anthropic factors that most contribute to the deterioration of cultural works are those produced by the emission of toxic gases emitted by vehicles in urban areas, whose particles can be deposited on surfaces, causing dark spots that end up conducing to aesthetic damage [58], as shown in a site placed in front of a museum (Figure 1). The constant emission of these products into the environment could turn out to be uncontrollable.

Various factors control the adhesion to the surface, mainly of a structural nature. That is why highly porous surfaces turn out to be more susceptible to the deposition of particulate material. Despite the variety of materials used in architecture and sculptures, their susceptibility to deterioration is different. While some materials deposit on the surface, others can penetrate through areas of weakness, leading to their delamination. Likewise, the effect of acid rain can cause constant damage to materials, which acts in the same way by precipitating mineral salts that contribute to the loss of consolidation and the modification of the surface [59,60,61]. Similarly, the emissions produced by proximity to industrial areas [62] can transport compounds that, when deposited on the surface, cause similar alterations to the surface [63], in addition to the increase in pathological processes such as cardiopulmonary disorders [64].

However, different factors must be considered in the interaction between contaminants and the exposed surface. For example, the mineralogical and textural composition of the material is one of the main factors determining the susceptibility to the entry of external material. Within the extrinsic agents, there are a series of pollutants of atmospheric origin from both natural and anthropic sources of pollution [65]. Atmospheric pollutants (SO_2_, NO_x_, CO_x_, CFC, CH_4_) and suspended particles [66], together with water, constitute some of the agents of deterioration that are more aggressive for stone materials, causing the degradation and alteration of minerals due to physical rupture and disaggregation, affecting the porous system [67]. The processes resulting from this interaction between extrinsic and intrinsic agents derive from degradation mechanisms such as dissolution, precipitation, recrystallization, or hydrolysis, involving mineralogical changes and textural properties of the materials and a physical loss of material and, therefore, of the historical and cultural value of heritage elements. A classic example is the emission of SO_2_ produced by vehicles, which, when in contact with stone surfaces of calcium or magnesium carbonates, such as those of buildings or sculptures built with limestone, reacts by generating crystallizations of gypsum [68]. This hydrated calcium sulfate confers a dark hue on the material. However, the action of SO_2_ is not exclusively on stone surfaces (walls of buildings, sculptures). It can also affect other essential pieces, such as museum objects of historical value. Among them, SO_2_ decreases the alkaline reserve in historical paper due to the uptake of the sulfuric acid formed, which depends on the local relative humidity. There is a strong interaction between diffusion, absorption, and the reaction of contaminants with the type of paper or the metallic ions of the ink, depending on the thickness and texture of the document [69,70].

To remedy this, nowadays, the use of consolidating [71,72,73,74] and self-cleaning products [75] based on nanomaterials is widespread, and its mission is to restore the lost cohesion to confer protection against the diverse aggressive agents [16]. However, the effectiveness of these products depends on the surface characteristics, properties, and product–substrate compatibility, and improper selection of the nanomaterials may cause irreversible damages after the treatments [75].

**Figure 2:**
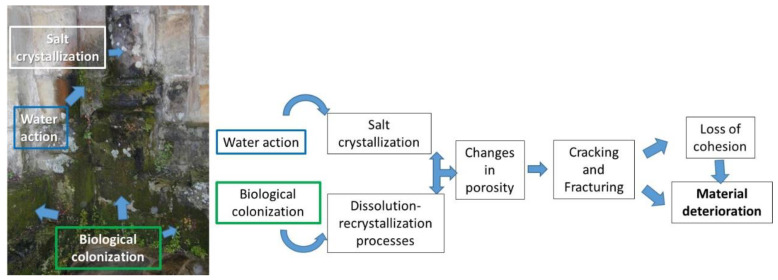
Architectonical monument deterioration by water action and biological colonization.

In addition to the anthropic factors, within the natural processes, two agents contribute to deterioration; one of them is the biological action, and the other is the water. Figure 2 shows one example of this behavior in a monument surrounding a lake, where humidity conditions and biological action cause changes in porosity and its consequent fracturing until the loss of cohesion. This is even more true when, throughout the year, temperature changes increase the water action on the surface.

Water is one of the primary agents of deterioration. Circulating through the porous surface dragging compounds that affect the material’s chemistry and react with its constituents is highly susceptible to causing additional damage. One of them is the action of cycles of freezing and thawing, in which changes in the state can give rise to stresses within the material, causing disintegration between grains, with a consequent increase in their porosity, finally reaching collapse [76]. The interaction between the rock’s minerals and highly soluble compounds dragged by the water, in specific conditions of pH or temperature, can generate the precipitation of salts. These salts generally occur in crystalline forms whose growth induces the fracturing of the surface, which in turn creates aesthetic modifications [77]. Quite evident examples are those pieces of underwater interest, highly deteriorated by their prolonged exposure to water and loaded with chemical compounds in the solution [54].

In the case of biodeterioration, the organisms adhere to the surfaces, helped by the porosity and roughness of the material [78]. These microorganisms can alter the material’s porosity [79] while providing chemical compounds that contribute to the precipitation of salts and therefore alter the texture and composition of the substrate. These new salts, such as those produced by polluting agents, crystallize inside the pores, generating tensions in the material to increase its deterioration. Likewise, organic and inorganic compounds can react with each other, leading to in situ phase transformation processes [80], which contribute to aesthetic and structural changes in the substrate [81]. Similarly, the action of plants on the surface of the monuments causes damage depending on the type of species. Its roots adhere to the surface, taking advantage of areas of weakness, which cause tensions that lead to the loss of cohesion of the substrate. Likewise, plants produce organic chemical compounds that, by occupying the pore spaces, easily lead to chemical reactions such as the extraction of calcium ions or other elements from the substrate, consequently accelerating the deterioration process [82].

Among many factors of deterioration, one of the most unexpected is that produced by the fire action in monuments or museum pieces. A rise in temperature causes the structure to break by heating, giving way to the alteration of the material [83,84,85,86]. The damage caused can be weighed with applications of coatings based on fire-retardant nanomaterials [87,88].

### Background of the Nanotechnology in Cultural Heritage Conservation

The application of nanotechnology for the conservation of historical heritage has evolved as its various properties, synthesis methods, and characterization equipment became known.

The beginnings of restoration work with nanomaterials started around 2001, led by the University of Florence Italy. In its first phase, the focus was the restoration of deteriorated pieces of pictorial value due to the aggressive action of polluting elements deposited on their surface, which can produce damage that may be irreparable [89]. Likewise, the same group restored ancient galleons attacked by water and chemical compounds through wood restoration. In the same way, successful results include restoring and conserving wall paintings of archaeological value that were highly affected by high humidity and sudden changes in temperature over the centuries [89].

The first generation opted for the use of simple materials, most of them oxides and hydroxides that, when reacting on the surface, managed to cover the material with a protective layer. This was when consolidants based on mostly metal hydroxides compatible with the substrate started to apply as the primary source in the restoration work [90,91]. These include the well-known alkali and alkaline earth metal hydroxides of Ca and Mg. It consists of modifying the porosity and filling the fractures within the material, applying nanomaterials with a similar composition to the substrate [92,93]. Nowadays, there is a constant interest in improving the efficiency of consolidant nanomaterials by trying different synthesis techniques [71,73,94,95] and studying the effects of specific physic-chemical and environmental conditions [96,97,98]. Another alternative was the use of water-based micelles and microemulsions (neat or combined with gels) for the removal of accidental contaminants and polymers used in past restorations of works of art and calcium hydroxide nanoparticles for controlling the damages produced by acidification in wall painting, paper, and wood [99].

Due to the success achieved in repairing and cleaning surfaces, many of them on canvases, sculptures, ancient wood, or architectural monuments, its objective was expanded towards a new challenge: how to control the effect of deterioration caused by biological action [100,101]. It was then time to design or apply nanomaterials already being used in medicine, such as nano-titanium oxide [100,102], nano-silver [103], or nano-Zn oxide [104] or nano-compounds mixtures [105,106,107,108]. The most frequently used biocidal nanostructured materials can produce a protective layer due to their photocatalytic power, favored by the specific properties of the nanomaterial [109]. This topic has continued to advance along the years with new ecofriendly strategies for controlling colonization on cultural heritage materials [12,110,111,112,113].

Fortunately, its effectiveness was relevant since nanomaterials with specific properties and combinations began to be designed in which the photocatalytic action and self-cleaning effectiveness would be the first objective to control [114,115].

In the same way, another possible risk, such as that caused by fire, needs more attention to prevent its deterioration [86]. It was the moment when materials such as magnesium hydroxide [116] or nanoclays [117] started to be used. For instance, in the event of a temperature rise, magnesium hydroxide acts as a fire-retardant protective layer [1]. The new generation of fire-retardants use combinations of different compounds such as magnesium hydroxide carbon nanotubes [118] or polymeric composites with traditional flame-retardants [119]. In many cases, the trend is looking for ecofriendly nanocompounds [120].

Considering that water is one of the primary agents of deterioration, the new goal was to apply waterproofing agents [95]. Among the possible candidates, silicon oxides in their amorphous variety began to apply by covering the surface with a hydrophobic protective layer [95].

As observed over time, the action of this type of nanomaterial needed reinforcement to make it more effective. In the case of SiO_2_, the trend was using mixtures with polymeric materials and gels with nanoparticles [121] that exert their action inside, causing in situ polymerization [122]. For instance, applications of silica nanoparticles and polymers on marble surfaces get to protect against water action, modifying the roughness of the deteriorated material [9,123]. However, the mixture of organic commercial products such as siloxanes with nanoparticles of SiO_2_, Al_2_O_3_, SnO_2_, and TiO_2_ may produce color changes and aesthetic damage, as reported in marble surfaces [124]. Nowadays, research about suitable polymers is a topic for improving their effectiveness and avoiding these damages [125]. Despite the pros and cons, new strategic nanomaterials can avoid additional damage on the surface [126].

The use of nanomaterial treatments gradually gave way to new trends, such as the beginning of composite materials with multifunctional properties. This was when materials with consolidating/fire-retardant or biocides/consolidating/hydrophobic properties began to be applied to solve several problems simultaneously [127]. Because of their interaction, the new mixtures gave rise to new compounds with different structures and stabilities.

New designs of nanomaterials started to attain specific properties, morphologies, sizes, and degrees of crystallinity more frequently, creating the need to evaluate these characteristics according to the environmental conditions, type of solvent, and concentration or reaction time. Synthesis methods that are bottom-up, such as sol-gel, hydrothermal, and colloidal, or breakdown, such as mechanical grinding or laser-based [128,129], began to interest researchers in achieving the most suitable treatment properties [94,130,131].

From this moment, the new stage of nanotechnology came to solve the problems in diverse fields. The construction field saw the opportunity to include nanomaterials for coatings isolative [132] and in the design of cement [133,134], concrete [135,136], or mortars [137,138,139] to improve their specific properties. Nanomaterials also began to apply to soundproof surfaces [140,141], thermal insulators [142], inhibitor coatings against corrosion [143], or salt attacks [144]. In addition, its application for restoring old stained-glass windows obtained better-quality glasses and ceramics [145].

The opportunity also came to museums, where paleontological pieces, sculptures, old paper books, and archaeological, anthropological, or cave art value pieces saw nanotechnology as a great ally. Specifically, the deterioration of paints due to the effect of salts or the accumulation of dirt onto the surface by airborne pollutants, which may conduce to aesthetic damages, required more effective cleaning [89,146]. Likewise, old ships started to be restored successfully [89].

Lastly, the new generations of nanomaterials are advancing vertiginously, coming to use nanocomposites [147,148,149,150], clay nanotubes [151], or single- and multi-walled carbon nanotubes [152,153,154]. Within the wide range of nanocomposites, protective nanocoatings include different compounds in specific combinations with consolidant properties for applications in stone (limestone, marble) [149], cellulose-based materials (papyrus, old paper, and wood) [150], or archaeological alloys [143].

Details about different nanocomposites with specific properties for the protection of different substrates [148] include, for example, combinations of Titanium oxides in mixtures such as TiO_2_-Paraloid 72 (Cu-Zn alloys pieces) [155], hybrid compounds of siloxane with nanosilica-siloxane (water repellent) [156], nano Ca(OH)_2_ (marble) [157], TiO_2_-SiO_2_ in the form of tetraethylorthosilicate (Theos) for marble [158], or TiO_2_-SiO_2_- polydimethylsiloxane, also known as PDMS (limestone) [159]. Likewise, their mixtures include combinations of ZnO-TiO_2_-Paraloid 72 (pottery), hydroxyapatite-Theos-PDMS for applications in sandstones, or silicon-based compounds with calcium oxalates for stone consolidation [147].

With all these advances, it has been possible to solve problems due to the advantages of nanomaterials, in which the increase in the surface area achieves better efficiency compared to those of a larger size and similar composition [160].

However, the effectiveness of these treatments requires considering various factors such as the application technique [161,162], concentration [163], time [164], temperature [165,166], or relative humidity [75,96].

Another concern is the deterioration of underwater interest pieces by their prolonged exposure to water, loaded with chemical compounds in the solution. Several treatments including nanomaterials such as TiO_2_, ZnO, and Ag nanomaterials dispersed in siloxane wax showed promising results against colonization in underwater marble, resulting in TiO_2_-Ag being more effective than ZnO nanoparticles [167]. There are alternatives, such as preparing bio-antifouling mortars, including Mg (OH)_2_ nanoparticles, to increase the resistance both in seawater exposure and laboratory samples [168].

The application of different nanostructured compounds with a tubular morphology, such as SWCN and MWCN, is an excellent opportunity for various cultural heritage applications. The unique hollow structure of CNT confers high mechanical, thermal and electrical conductivity (bulk resistivity ~3.8 × 10^−4^ m in a CNT sheet). Moreover, CNTs are chemically stable because their carbon atoms form sp2 covalent bonds in the form of a honeycomb [169].

Among their mechanical properties, their high tensile strength/stiffness, which is better than that of any metal, stands out [170]. The elastic resistance is superior, so CNT can be bended, twisted, kinked, and buckled without damage [170]. Other advantages are the thermal conductivity of MWCNT being higher than that of SWCNT, both exceeding the diamond. MWCNTs thermal properties are similar metallic properties which confer better thermal properties when included in the polymer composite [169].

They also have high hydrophobicity and high protection against photo-degradation depending on the number of walls, which are helpful in the absorption process of other nanomaterials [153].

Nowadays, the industry takes advantage of these properties for manufacturing photocatalysts with thin layers of MWCNT, which is an opportunity for conserving works of art [152]. The remarkably light and robust properties and their electrical conductivity turn out to be beneficial for creating efficient heat surfaces to quickly guarantee ultra-stable temperatures for large surfaces and short heating and cooling times. Thus, the research focuses on innovative and highly accurate mild and flexible heating devices for conserving various pieces of cultural heritage [171].

Furthermore, advances in synthesis techniques include graphene for wall painting [172] and stone protection [173]. Diverse synthesized routes allow for obtaining different shapes including tubular nanotubes, nano-rods, nano-needles, or nanowires of different compounds such as titanium oxide [174] or Zn oxide [171]. In the case of Zn Oxide, the combination with silver has managed to progress in the field of photocatalysts with biocidal action [153,175]. The new fire-retardant generation includes carbon nanotubes mixed with different nano-compounds with fire-retardant properties. Among them, nowadays, the most common combinations are nanocomposites with metal oxides, MWCNT-nanoclays [176], graphene, sepiolite nanorods, nano-cellulose, fullerene [176], or CNT-magnesium hydroxide [118].

There is a broad potential in using CNT to protect museum pieces, such as photographs [177], based on bio-inspired applications, such as applying the mechanism that geckos (reptiles) have on their feet to adhere firmly to surfaces. This property is due to the elastic beta-keratin nano-hairs on their feet and toes, which collectively generate a strong enough van der Waals force to hold the animal to an opposing surface while simultaneously disengaging at will [178].

Within the field of dry adhesives, the new trend is using this type of structure. When applying this type of product, it is possible to achieve strong adhesion in the normal and shear directions under stress, with low peel resistance, which offers the possibility of easily removing it from fragile surfaces during treatment and using it as a mounting adhesive [177].

Studies have been conducted on vertically aligned carbon nanotubes that resemble adhesive hairs on gecko feet, with additional superior mechanical, chemical, and electrical properties, proving to be a promising candidate for advanced fibrillar dry adhesives [178]. However, its effectiveness depends on the packing density and the roughness of the vertically aligned CNT surface [179]. Research in this regard emphasizes that increasing the roughness of the matrix surface strengthens adhesion in the normal direction but weakens it in the cutting orientation [179].

The interest in controlling the microclimatic conditions in different places with heritage value is a topic that has progressed over the years. Among the diverse techniques, hygrometry and infrared thermography result in no invasive procedures for controlling moisture, which is very useful in conservation procedures [180]. Advances in incorporating sensors capable of detecting changes in local humidity and temperature have allowed for controlling their action in architectural and archeological sites and museum pieces susceptible to deterioration [181]. Nowadays, another application of nanotechnology in museums is to control corrosion produced by gaseous corrosive agents existing in the indoor environment due to the combustion of fuel fossils, nitrogen oxides, plants, or automobiles [182],—for instance, the use of resistive gas sensors to detect NO_2_ made up of single-walled carbon nanotubes mixed with ZnO (SWCNT/ZnO) deposited on a sapphire substrate. Investigations report that this configuration has high stability but that NO_2_ detection highly depends on the microstructure’s changing matrix and the composite material’s preparation conditions [182].

## 3. Risk of Toxicity during Handling with Nanomaterials in Conservation Procedures

One of the main risks when working on heritage conservation is the one that occurs due to handling products that include nanomaterials. There are three modalities: spray, brush, and immersion [161,162]. In the last case, when the piece is restored, it can be moved from the site and placed on a solution rich in nanomaterials for the impregnation by capillary rise. However, among the mechanisms of entry into the body, it is essential to consider the high risk that occurs during application by spraying these nanomaterials [183,184,185]. The inhalation route is the body’s most exposed part in this procedure. However, the possible risk of access through the skin is not ruled out [186,187], mainly on the face, hands, and arms during routine treatment, in addition to admission through the eyes due to a possible splash or through the ear when the dispersion in the environment is high [188,189]. Exposure using the broaching technique is also crucial to consider. In this procedure, the nanomaterials remain on a brush in the solution, and contact with the skin can occur accidentally. That is why it is essential to take the appropriate protection measures, such as gloves, which, as described below, must comply with the regulations, be highly resistant, be not very porous, and have resistance to contact with liquid solutions (aqueous, alcoholic, or gels) that can eventually react with the glove, exposing the skin. In addition, since nanomaterial solutions remain in the brush, any contact with surfaces in the laboratory or the handling area leaves the nanomaterial exposed, which ultimately increases its risk of dispersion to surrounding places, including the hazard for the personnel, living beings, or the environment.

Another means by which there is a high risk of internalization is during cleaning procedures due to the release of nanoparticles into the environment and the operators’ exposition to the dust emitted. In the same way, it happens in techniques for synthesizing nanomaterials, in which the handling of both reagents and the particulate nanomaterial leaves the operators highly exposed, in addition to the people in charge of waste management and those who are in the same work area. These risks increase during the synthesis by breakdown methods, which minimize particles by mechanical grinding [190,191] or laser techniques [192].

## 4. Interaction of Nanomaterials with the Human Body

The dispersion of nanomaterials in the environment can affect the ecosystem because the particles can travel through the air and deposit in water and soils, affecting different living beings, such as aquatic and terrestrial organisms, fauna, and microbiota [193]. Environmental studies indicate the necessity of analyzing parameters that could affect nanoparticles’ properties and their toxicity, such as transport or movement mechanisms in air, land, and water and their diffusion capacity (e.g., aerodynamics, filtration in porous media such as soil, dissolution/dispersion in aqueous media), agglomeration, wet and dry deposition, or their gravitational properties [194].

Just as they can cause environmental contamination, they can also be incorporated into the human body and animals due to their fine size [195,196]. There are several routes of entry, the main ones being ingestion, aspiration through the respiratory system, tear ducts, ear canals, or skin contact [197]. For these reasons, the risk differs depending on how it is applied. For example, in the case of consolidation or protective treatments, using a brush or spray might generate different particle emissions, which can enter the body depending on the type of exposure.

### 4.1. Critical Particle Properties

According to studies to date endorsed by international organizations on toxicity such as the European Commission [198], several parameters are crucial when analyzing toxicity. The main one is the particles’ size, which is greater than the smaller size. In addition, currently, another criterion to consider is the morphology of the particles, including the aspect ratio [199], the flexibility, the degree of agglomeration/aggregation [200], and the effective surface area [198]. Other critical parameters are the chemical composition and solubility [201]. Regarding this last point, it is necessary to consider the persistence and analyze its water solubility, whether high, medium, or low, and its resistance to breaking. Furthermore, it is essential to consider its dustiness degree, referring to the developed dust that remains in the air, analyzing whether it can be high in the case of fine dust, medium in the case of crystalline particles, and low for non-friable solids or pellets [198].

On the other hand, based on the data reported about the toxicity of nanomaterials, it is essential to analyze aspects in more detail. For example, critical studies insist that there are a series of criteria to consider when assessing toxicity risks depending on the exposure, dose, bioavailability, bio-persistence, bioprocessing, bio-modification, and bio-clearance of nanoparticles or nanofibers [202]. Studies even criticize the omission of some critical considerations, leading to misinterpretations and, thus, contradictory results due to poor nanoparticle physicochemical properties characterization in both in vitro and in vivo tests [201,203]. Therefore, there is a necessity of broadening the research about the effect of different specific parameters on the nanomaterials’ toxicity degree, as, in many cases, it may give rise to partial conclusions.

#### 4.1.1. Particle Properties

The particle size, morphology, and specific surface area are the main criteria considered that contribute to its toxicity in the same way as its degree of agglomeration or aggregation. However, when referring to “agglomeration” and “aggregation,” it is essential to note that both terms’ assumptions are indiscriminately used, which leads to confusion. According to the European Commission 2013 [198], agglomerate means “a collection of weakly bound particles of aggregates where the resulting external surface area is similar to the sum of the surface areas of the individual components”, and aggregate means “a particle comprising strongly bound or fused particles.” On the other hand, the British Standard regulation of 1991 refers to an agglomerate as “an assembly of particles rigidly joined together as by partial fusion, sintering or by growing together” and to an aggregate as “an assembly of particles which are loosely attached to each other” [204]. Subsequently, the UK NanoSafety Partnership Group (UKNSPG) assumed the classification of the European Commission [205]. Many authors refer to the old British concept [206,207], while others prefer the European one [208].

For practical purposes, the possibility of emitting particles into the environment depends on the cohesion degree between the primary particles. It is crucial to consider that aggregated/agglomerated nanoparticles behave differently in transport and reactivity with the environment depending on their own characteristics. Depending on the compaction degree and the specific properties of the primary nanoparticles, their toxicity may increase when released into the environment [207]. In this sense, a series of forces govern the particle interaction known as van der Waals (vdW) attractive and double-layer electrostatic (EDL) [207]. This behavior can generate these forces when handling powders, resulting in caking, lumping, or the local accumulation of electrostatic energy [209]. Models of the interaction of particles in suspension conclude that the sum of the vdW attractive and EDL forces determines whether the particles adhere or repel. In this sense, is is essential to consider the attachment efficiency, also known as the sticking coefficient, which consists of the probability that two particles attach [207] and can be explained by an Ostwall ripening process where nanoparticles tend to grow at the expense of smaller particles, increasing their dimensions [210]. Furthermore, the large specific surface area makes nanoparticles have a lower thermodynamic stability and tend to agglomerate over time because of their high free surface energy compared to that of larger particles [210]. However, the conduct of suspended particles is dependent on the shape, size, composition, structure, or macromolecules [207]. Based on these factors, the emission of particles into the environment could change and, therefore, the risk of toxicity could too. In addition, it is necessary to consider the relationship between the tensile strength and packing density [211]. Experiments regarding agglomerates of titanium dioxide and black carbon powders found that the vdW force can be modified depending on the existing water on the particle surfaces in the form of adsorbed layers or liquid bridges [211].

Reports about the agglomeration of nanoparticles and their effect on health indicate how the state of agglomeration can cause diseases. For example, internalized silver nanoparticles can lead to thrombus formation in blood vessels, which leads to thrombosis [212,213]. In addition, other authors focus on its impact on the ecosystem, pointing out how the increase in the agglomeration of silver nanoparticles can decrease their ecotoxicity [208]. On the other hand, studies on agglomerated titanium oxide nanoparticles indicate that the size of the agglomerate influences DNA damage, it being more significant when its size is more prominent. The same authors emphasize the need to establish a protocol during the solution preparation procedure, insisting on increasing the sonication time to reduce the size of the agglomerates [214]. In the case of carbon nanotubes, studies indicate that a high level of agglomeration results in inflammatory processes such as those observed in the spleen and liver [215].

Depending on the synthesis method, the particles present different specific properties and, therefore, differences in the behavior during the internalization process. Likewise, each morphology has unique free energy compared to alternative forms in a constant ratio of surface to volume [216]. Criteria such as the type of solution (aqueous, alcoholic, concentration), the pH of the medium inside the body, and the solubility of each one of the chemical compounds condition their internalization and can vary significantly. Many works report the formation of several shapes, such as spheres, rods, flower-like shapes, cubes, plates, shells, or chiral geometries, which affect, in different ways, both the living beings and the environment, depending on the synthesis method [217].

With the advancement of research in the field of toxicity, there is a tendency to study dependency morphology. For instance, within the different nanocomposites used in conservation, studies on nanosilver analyze how morphology affects the degree of toxicity. Studies using different silver nanoparticles confirm that the degree of toxicity depends on the morphology. Thus, toxicity could be higher in the ionic state, followed by spherical particles, being lower and similar between cubic and prismatic particles [218]. Particles with a low aspect ratio could be more toxic than particles with a high aspect ratio [218]. However, it can not only be generalized that the toxicity of spherical particles is more significant, considering that many of them come from the aggregation of primary nanoparticles with different morphologies that form spherical superstructures [216]. Depending on the synthesis method, they can result from the aggregation of nanorods, ovoids, or nanofibers. In this sense, it is possible to obtain spherical shapes by the aerosol route of Ti oxide [174], nano SiO_2_ [219], or Zn oxides from sol-gel [220].

Considering that the particles with the lowest aspect ratio are those that are spherical, within this type, a comparison between iron nano oxides indicates that rod-like particles are more toxic than spherical ones [221]. In the same way, studies on nano-hydroxyapatite found that plate- and needle-shaped nanoparticles caused the death of a higher proportion of cells than spherical and rod-shaped nanoparticles [222]. Changes in the morphology and particle size lead to a change in the surface area and, therefore, in their degree of toxicity [221].

In addition, some studies talk about controversies in the toxicity assessment, pointing out the importance of analyzing the aspect ratio, taking into account its dependence length, as reported in studies of the nanotoxicity of TiO_2_ nanofilaments [223]. Specifically, nanobelts’ assessments indicate a higher degree of toxicity in TiO_2_ particles > 15 μm than in smaller ones [224].

Many studies discuss the possibility of developing different morphologies depending on the precursors and experimental conditions using the same synthesis method. For example, nanostructured ZnO particles obtained by hydrothermal synthesis can have different aspect ratios, particle sizes, or surface areas, developing shapes such as nanoplates, nanorods, tubules [225], or nanowires [226]. Similarly, by modifying the manufacturing routes, it is possible to obtain a wide variety of morphologies, as reported in nano-hydroxyapatite (Calcium-phosphate hydrate) [227]. Therefore, toxicity may vary depending on its specific characteristics.

Other cases are essential to analyze, such as applying nano alumina additions in Paraloid B72 coatings on old metal substrates, which has been proven to be effective for their protection against processes that generate the material’s corrosion [228]. However, it is crucial to consider that its cytotoxicity may vary according to the morphology [229]. For example, a possible emission of alumina during handling can differ depending on its specific properties. For instance, spherical particles can reach the brain, thymus, and lungs, depending on the dose. At the same time, smaller or larger nanorods can occupy the liver, kidney, heart, lung, and thymus. Furthermore, long aluminum nanorods can induce a stronger inflammatory response than short nanorods [229].

#### 4.1.2. Differences in Surface Roughness

Among the morphological properties of nanomaterials, apart from the shape and size, one of the properties that most influence their toxicity is the roughness of the surface. For example, spherical particles can have a smooth or rough edge that will intersect with the cell depending on the roughness. For example, some studies analyze how flower-like shapes have more toxicity than those with smooth-edged spheres, causing cell membrane disruption, as reported in human endothelial cells [230] However, there are differences in damage depending on the termination of the sharp surfaces. In this sense, it is crucial to consider that particles can develop corners with rounded edges depending on the specific manufacturing process or undergo a change internally during the dissolution process due to interaction with body fluids. This is the case of rod-like particles, whose toxicity is lower than that of the prism, sphere, or needle shapes, as observed in several studies [231,232].

#### 4.1.3. Nanofibers

Nanofibers are one of the cases in which toxicity could be more aggressive in a similar way as asbestos. As explained above, its degree of toxicity depends on its aspect ratio. However, the fibers’ internalization, regardless of their composition, can be randomly distributed so that they generate a new roughness according to the different orientations of the fibers [233]. Neurological studies explain that nanofibers can affect cell generation and modify their growth patterns, as observed in neural stem cells [234]. The nanofibers’ disposition can affect the neural stem cell elongation and neurite growth in the aligned nanofibers’ direction [234]. In particular, nanofibers mimic the structure of the fibrous components of the neural extracellular matrix [234]. However, the aggregation is sometimes favorable for reducing the toxicity of individual fibers depending on the local environmental conditions or the assembly in which they are supported, such as those used in reinforcing polymeric matrices that, with the degradation process, can release fibers towards the environment, affecting living beings and the ecosystem [235]. Among the factors, it is essential to consider both flexibility and rigidity. Although the fibers are more or less flexible, they can access the respiratory and circulatory systems of animals and humans through different routes. Thus, fibers and particles can obstruct the air passage and reach the blood, which causes thrombi, leading to cardiovascular or lung diseases [236]. However, in the case of fibers, rigidity is a crucial parameter that controls their degree of aggressiveness. Typical cases are the risks of exposure to SWCNT or MWCNT, which pose differences in their rigidity, SWCNT being less rigid and more easily aggregated [237]. Nevertheless, new advances in research about SWCNT/MWCN toxicity insist on the need to mitigate its hazard during the manufacture, handling, and applications of these materials [237].

#### 4.1.4. Physical Chemical Properties

One factor that significantly influences nanomaterials’ toxicity when internalized is their chemical composition. The local reaction depends on a series of factors inherent to the local pH of the internal environment or the surface charge of the nanoparticles, the positively charged ones being more toxic since they enter cells more quickly than the negative and neutral ones [238]. In this sense, it is essential to keep in mind the difference in the ionic solubility of the different compounds, which change according to the dispersion media properties being different between aqueous and organic solvents [239]. There are other parameters to consider regarding the solubility of inorganic nanoparticles, which tends to increase in acidic solutions, such as gastric fluids [201]. The recommendation of experts pointed out that their solubility should be essentially checked for toxicity evaluation following oral exposure [201]. According to the reports, metallic silver and insoluble silver compounds are more toxic than soluble compounds of the same composition [240]. The solubility between SiO_2_, ZnO, and TiO_2_ is different, so TiO_2_ is insoluble, followed by SiO_2_, unlike ZnO, which is highly soluble in weak acids and makes it very susceptible to corrosion [241]. However, in the case of TiO_2_, the solubility of the main polymorphs (rutile and anatase) is different. Experimental studies report that the rutile solubility in water is relatively low compared to that of anatase at 20–320 °C [242], but anatase is soluble in hydrofluoric acid (HF) [243,244]. Therefore, it is expected that, when coming into contact with different non-aqueous solutions due to internalization, the behavior may vary depending on the structure of each nanostructured compound and the type of acid solution.

Furthermore, specific studies carried out on tubular cells identified that solubility is one of the factors that can affect them to a greater or lesser degree, as was observed in comparisons between nanoTiO_2_, ZnO and CdS, being more significant when the solubility was higher, as observed in ZnO and CdS, which led to destabilization in the lysosome [245].

As reported by studies, ZnO is highly soluble and concentration-dependent, and a dose increment facilitates the release of Zn^+^ with the subsequent direct or indirect induction of oxidative stress [246]. Therefore, it is necessary to consider that the toxicity will be different.

#### 4.1.5. Structure and Defect

The degree of structural disorder is the least considered aspect when evaluating the toxicity of nanomaterials, it is necessary to consider that, due to the different types of synthesis and experimental conditions, there may be different degrees of disorder in the nanomaterials obtained. An example is that of Mg(OH)_2_, which depends on the synthesis method, such as sol-gel, hydrothermal, or colloidal routes. They can generate differences in morphology, preferential orientations, particle size, and homogeneity [247]. Even in hydrothermal-type or solvothermal processes, a change in the concentration of the reagents can lead to a high degree of defects [247]. The degree of defects (vacancies, stacking faults, twins, or dislocations) favors crystallization or surface roughness development, affecting cells. Few articles stop analyzing defects as factors that increase toxicity; however, studies report the toxicity of SWCNT or MWCNT [248], In addition, observations made in silver compare the effect of morphology, indicating that the plate-type forms developed more defects, unlike the spherical or nanorod forms [249]. However, it is impossible to generalize in this case, since analyzing the specific synthesis conditions used and the subsequent heat treatments is crucial. Other results talk about the existence of defects in complex nanoparticles of TiO_2_ formed by rutile-anatase-brookite, which alter the surface charge affecting its environmental behavior [207].

Furthermore, when the nanoparticles have several phases with different symmetries or compositions, the stress generated at the phase boundaries produces dislocations, which ultimately modify the particle’s compaction and the external surface. One of the most cited cases that have given rise to conflict is interference between the rutile and anatase phases. Some authors maintain that anatase is more toxic than rutile [250], and others maintain the opposite [251]. In these cases, it is necessary to analyze the structures and the degree of disorder at the atomic level, the presence of defects, and the subsequent modification of their surface. It may be the most forgotten among all the toxicity measurement parameters.

### 4.2. Deposition Mechanisms of Nanomaterials

The interaction of nanomaterials with the human body has similarities with other processes, which facilitate the understanding of their behavior. For example, within them, it is possible to associate the human body with its different organs, tissues, or cells as if it were an ecosystem. Nanomaterials behave differently in an acid or a local alkaline environment, depending on the specific characteristics, affecting the nanoparticles so that they will continue to transform inside them before and after the cell phagocytosis. Furthermore, primary nanomaterials behave like gases, have a rapid diffusion capacity, travel long distances, and show low sedimentation rates [252]. Once nanomaterials have entered the airways, they are deposited based on different deposition mechanisms. Diverse access routes to the organism will reach the different organs and interact locally with the different tissues or cells. The access routes are nasal, olfactory, ophthalmic, oral, and dermal [253].

The following section explains the main effects that can occur from continuous or eventual exposure to nanomaterials during routine procedures in conservation work. Table 1 summarizes the access route and internalization mechanisms into the human body.

#### 4.2.1. Access through the Nasal Route

**Figure 3:**
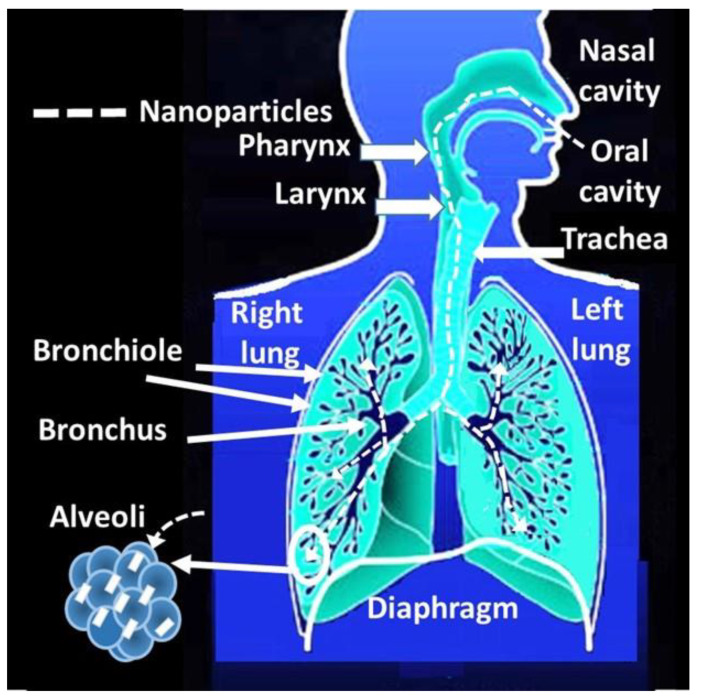
Nasal access of nanomaterials to the respiratory tract.

The nasal route stands out among all the access routes to the body. Once the particles enter the nasal passage, they reach the respiratory tract and the brain through the olfactory nerves (Figure 3). However, understanding the mechanism of entry into the body through the respiratory tract has raised a great challenge over the decades. The particle size stands out among several critical parameters. According to the studies, the mechanisms responsible for the deposition of particles in the pulmonary airways during the inspiratory phase of a breath at tidal volume are diffusion, interception, impaction, and electrostatic attraction [254,255,256]. However, there are differences between the diverse input mechanisms depending on the particle size. For example, the transport of microparticles depends mainly on inertial impaction and sedimentation, while that of nanoparticles depends mainly on diffusion [257,258,259,260]. Specifically, the mechanisms of deposition by the inhalation of aerosol particles smaller than 10 microns are inertial impaction, gravitational settling, and diffusion [192,254,261]. Coarse particles (>3 μm) mainly deposit by impaction due to abrupt changes in the direction of the airflow that occurs in the mouth (or nose) and the upper respiratory tract, including the pharynx, larynx, trachea, and bronchial region. Gravitational settling is most efficient in the narrow, randomly oriented ducts and air spaces further down in the lungs (bronchiolar and alveolar region) [254].

Electrostatic deposition in humans and animals is common from aerosols, which generate considerable charge depending on the particle size [262,263]. For instance, many industrial processes generate electrostatically charged nanoparticles [264], which increase the number of inhaled particles deposited in the lung. Moreover, the electrical charge is the key to understanding the behavior of nanoparticle aerosols and their effects on health [265]. Its attraction occurs due to the opposite charge between the particle and the respiratory tissue surface. In this sense, the charge–mass relationship of the particle governs the strength of this attraction, which is most effective when the particle size decreases. For example, in the human respiratory system, based on the International Commission on Radiological Protection, ICRP 1994 model [266], it was possible to determine that for the atmospheric nanoparticles with a size between 6 nm and 30 nm and which are negatively charged, the Brownian deposition mechanism predominates [267]. Likewise, a change in the polarity of the nanoparticles—for example, from 16 nm to 30 nm—significantly increases alveolar deposition in terms of surface area. This increase maintains a plateau of up to 150 nm [267].

**Figure 4:**
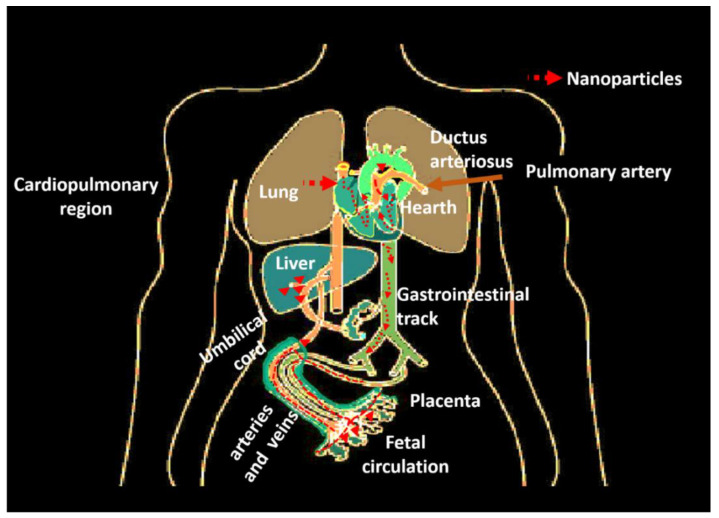
Details of the cardiopulmonary region showing how nanomaterials cross the alveolar-pulmonary epithelium and reach the interstitial zones, reaching the circulatory and lymphatic system, distributed throughout the body. In pregnant women, some nanoparticles can reach the placenta and affect the fetus.

**Figure 5:**
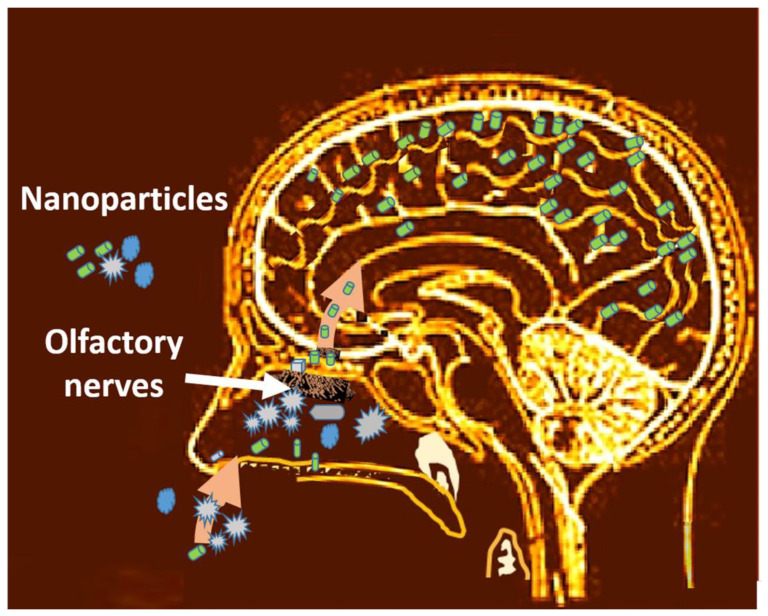
Access of nanoparticles to the brain through the olfactory way.

Translocation is the mechanism of entry into the body of nanomaterials [268]. These can cross biological barriers and reach areas of the body other than the entry route, depending on their solubility [269]. For instance, in the case of the inhalation route, they cross the pulmonary alveolar epithelium, reach the interstitial areas, and reach the circulatory system, distributed throughout the body [270] (Figure 4). Another example is that the nanomaterials captured at the nasal level can access the brain via the olfactory nerve (Figure 5).

Although the electrostatic charge plays a crucial role in the internalization of nanomaterials through the respiratory tract, translocation is the dominant factor in the dispersion to other organs, such as the circulatory system, the brain, or from the lungs to the blood [268].

There is interest in understanding the mechanisms of translocation to other organs. While extensive studies focus on understanding the immediate consequences of exposure to these materials, the long-term effects of potential translocation to secondary and even tertiary organs still need to be better understood [271]. Advances regarding the toxicological effects in secondary organs report oxidative stress, inflammation, cytotoxicity, and the dysfunction of cellular and physiological processes [272]. In this sense, it is essential to consider specific characteristics of nanomaterials such as the charge, size, lipophilicity, or protein absorption [268]. However, as knowledge on the subject advances, conflicts begin to appear, which some authors highlight, insisting on the need to delve into the physiological impact [268]. In addition, others emphasize the need to develop in vivo and ex vivo models to know the relationship between the structure of nanomaterials and the penetration capacity [269]. However, according to the experts, current in vitro models have advantages and disadvantages, making it difficult to address nanomaterials’ interactions with various biological barriers [269].

Specific cases carried out in the alveolar area comment on a high degree of vulnerability due to the absence of mucociliary clearance and a fragile air–blood barrier, which can facilitate the translocation of particles to secondary organs [254]. However, its movement also depends on the particle’s properties. For instance, it is not the same if the particles are fibrous since they can be oriented along the respiratory tract until they collide with the bifurcations, where they will finally be deposited, obstructing the passage of air [273]. In this case, the fiber length may be the predominant metric determining the toxicity of bio-persistent fibrous nanoparticles [274]. This occurs with the carbon nanotubes (CNT) and carbon nanofibers (CNF), whose dimensions and high aspect ratio could be similar to those of the highly pathogenic asbestos fibers [275].

Several deposition models endorsed by the International Commission on Radiological Protection (ICRP) and multiple-path particle dosimetry (MPPD) report the dependence of the particle size on the deposition region [276]. For example, the deposition of particles with a particle size between 5 and 7 nm would predominate in the bronchial region, 10–50 nm in the alveoli, 1 nm (80%) in the nasopharyngeal, or 8–10 microns would deposit in the extra-thoracic and 75% on the nasopharyngeal region. However, there are intersection zones at 300 nm or 1 nm. Particles larger than 300 nm would have a lower probability of deposition in the bronchial region. In the case of 1 nm, 80% would correspond to nasopharyngeal, 20% would correspond to the bronchi, and 0% would correspond to the alveoli [276]. However, in all these models, it is crucial to consider how the intrinsic toxicity can be affected by agglomeration and by the aggregation size [199]. For instance, singles or agglomerated particles could affect the pulmonary pathways differently, generating inflammatory reactions after intra-tracheal instillation [200].

Although many of these models are semi-empirical, it is crucial to consider other experts’ opinions that parameters such as the breathing pattern, particle characteristics, flow dynamics, and morphological structure affect pulmonary deposition, including age, sex, and health status [254].

However, among the particle properties, it is crucial to consider parameters such as relative humidity or temperature because the inhalation of particles changes according to dry or wet environments [254].

#### 4.2.2. Access to Circulatory and Cardiovascular Systems

Once the nanoparticles enter the respiratory tract, they can reach the gas exchange zone, where the air–tissue–blood barrier between the alveolar wall and the capillaries is thin (Figure 4). At this site, there are greater possibilities for nanomaterials to reach the blood through the alveoli, which, within the different parts of the respiratory system, are the most exposed to environmental exposure, unlike the bronchi, which have a mucociliary layer that eliminates particles deposited in the lungs [197]. In addition, the nanoparticles can also translocate to the lymphatic system and distribute along the body organs by both systems (Figure 4). In the case of pregnant women, they can reach the placental barrier and cross it with the risk of affecting the fetus’s brain and modifying its DNA [197]. When the nanoparticles reach the cardiovascular region, they can alter vascular endothelial cells, thus affecting the dynamics of vascular tone, impairing endothelial function, and finally affecting the hearth, with risks of myocardial infarction, hypertension, arrhythmia, and thrombosis [277,278].

#### 4.2.3. Access to the Brain through the Olfactory Way

Within the translocation mechanisms, it is crucial to mention the case of nanomaterials that reach the brain pathways. The olfactory nerves perceive the nanoparticles on the outside that run towards the interior of the neurons (Figure 5). This accumulation can have significant neurosensory consequences, leading to diseases such as Alzheimer’s [279]. For example, iron- rich particles from vehicle emissions and those produced in industrial processes can lead to oxidative stress. This type of nanoparticle dispersed in the air due to pollution causes brain injuries primarily in dogs due to their high content of olfactory nerves [280].

#### 4.2.4. Access through the Eyes and Tear Ducts

**Figure 6:**
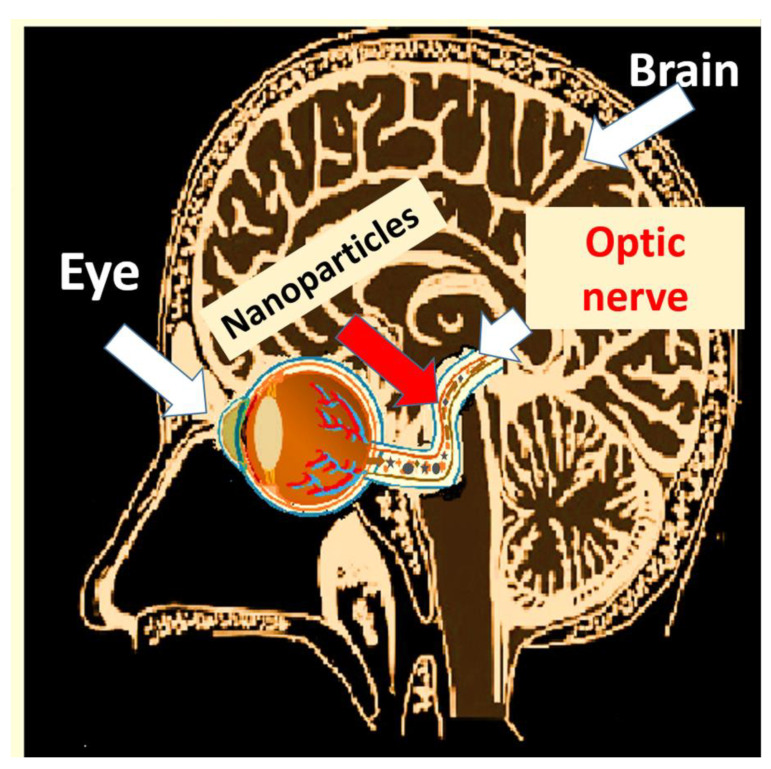
Entry of nanoparticles through the eyes and tear ducts and subsequent displacement to the brain through the optic nerve.

The incorporation of nanoparticles into the brain can also occur through the eye (Figure 6). Different irritating nanoparticles, in addition to affecting the different parts of the eye (iris, macula, retina, lens), gain access through the optic nerve. For instance, silver nanoparticles or multiwall carbon nanotubes (MWCNT) could cause increased cell apoptosis and oxidative stress. Experts point out that iron nanoparticles could cause retinal detachment, internal bleeding, and age-related macular degeneration. Similarly, exposure to ZnO could cause an increase in retinopathies [281]. In the case of silver and TiO_2_ nanoparticles, they could translocate into the central nervous system though eye-to-brain pathways, which could induce neuroinflammation [282].

#### 4.2.5. Effects of Nanoparticles in the Eyes

Studies carried out by experts point out the importance of keeping in mind the effects of the contact of nanomaterials with the eye, pointing out that although there are barriers that prevent the access of materials to the eyeball, the smaller the size of the nanoparticles, the greater the contact with the ocular surface [281]. According to the experts, the main area where nanomaterials deposit is in the cornea, where they remain for a longer time, depending on their specific characteristics, exceed the ocular surface’s barriers, and reach the retina [283] and the eye’s posterior segments (Figure 6). A size dependency determines the speed and quantity of nanomaterials susceptible to internalization through this route. Specifically, the migration through the epithelial barrier can cause cytotoxicity and inflammatory responses [284]. These same nanoparticles can induce cellular toxicity and a systematic immune response that also affects the lens, the retina, the optic nerve, and the macula [284]. Therefore, it is essential to consider that the internal contact between the tear duct and the nasal cavity is as dangerous as if the incorporation were through the nose. Thus, it could finally reach the brain and the central nervous system [285], with the consequent internalization through the different routes as it happens when they access the nose. For instance, recent compilations about access to metallic nanomaterials highlight how eyes are vulnerable to zinc oxide, silver, or TiO_2_ [285]. In the cases of TiO_2_ nanoparticles, the same authors report damages such as apoptosis induction, cell growth inhabitation, and induced excessive ROS generation, which ultimately led to irreversible cell damage and death [285,286].

#### 4.2.6. Oral Access and Gastrointestinal Region Interactions

**Figure 7:**
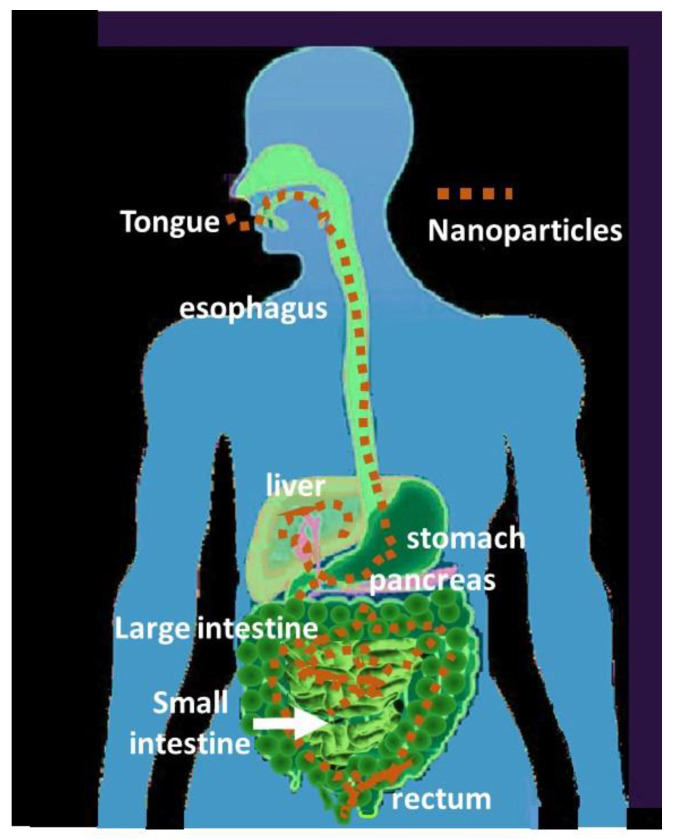
Access route of nanomaterials through the gastrointestinal system.

The gastrointestinal access route is the one with the highest internalization risk (Figure 7). However, the differences in the solubility of nanomaterials and the effect of pH are criteria for determining their dispersion and incorporation into different cells. A detailed study of the effects on the gastrointestinal tract [287] highlights that, for example, the pH is lower in the stomach (7–8) compared to in the small intestine (6.5–7) and the colon (7–8). The same authors comment on the various factors, highlighting the dependence on the size and charge of the nanoparticles, which condition their translocation, leading to Chron’s disease, ulcerative colitis, and cancer in extreme cases depending on the dose and composition of the nanomaterials [287]. For example, TiO_2_ between 25 and 80 nm causes inflammation in stomach cells. In the case of the ZnO particles, they point out that ingestion produced intestinal obstruction due to the aggregation of the particles and intestinal and stomach inflammations. In the case of nano SiO_2_, the toxicity of amorphous nano SiO_2_ is less than that of crystalline. There is more significant contact between the particles and the cytoplasm, when the organelle membranes of the epithelial cells of the esophagus rupture. However, it is necessary to deepen the effects of SWCNT and MWCNT [287].

##### Access to the Liver

The liver stands out among all the organs for being the body’s detoxifier. Nanoparticles interact with liver cells, modifying their structure and functions (Figure 7). The reports indicate microhemorrhages and severe necrosis caused by silver; liver inflammation due to the cellular infiltration of TiO_2_ by oxidative stress due to mitochondrial changes and fibrosis; and karyopyknosis due to ZnO, leading to apoptosis and necrosis due to irreversible chromatin condensation in cells. In all cases, its risk is cumulative and dependent on the concentration [288].

#### 4.2.7. Access to the Urinary Track

**Figure 8:**
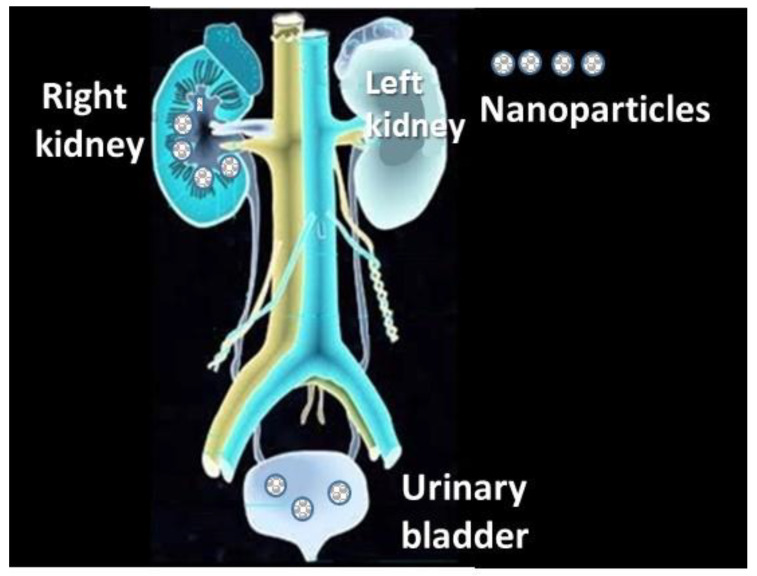
Accumulation of nanomaterials in the urinary track.

The kidneys are among the most affected organs since the nanoparticles from the blood supply are eliminated, being very susceptible to toxic metals (Figure 8). However, their accumulation depends on the nanoparticle size, the surface, and the type of cell they access (glomerular or tubular) [289]. In addition, metal particles can increase the formation of reactive oxidative species (ROS), producing oxidative damage, the induction of antioxidant enzymes, and apoptosis [289], which affects different cells depending on the specific solubility of the metal [245].

In all cases, the toxicity increases depending on the concentration of the nanomaterials and their solubility [202,290].

There is a high risk of forming urinary calculi due to the supersaturation, which favors the crystals’ nucleation, being equally feasible with their aggregation and continuous growth until they form stones [291].

#### 4.2.8. Access through the Skin

**Figure 9:**
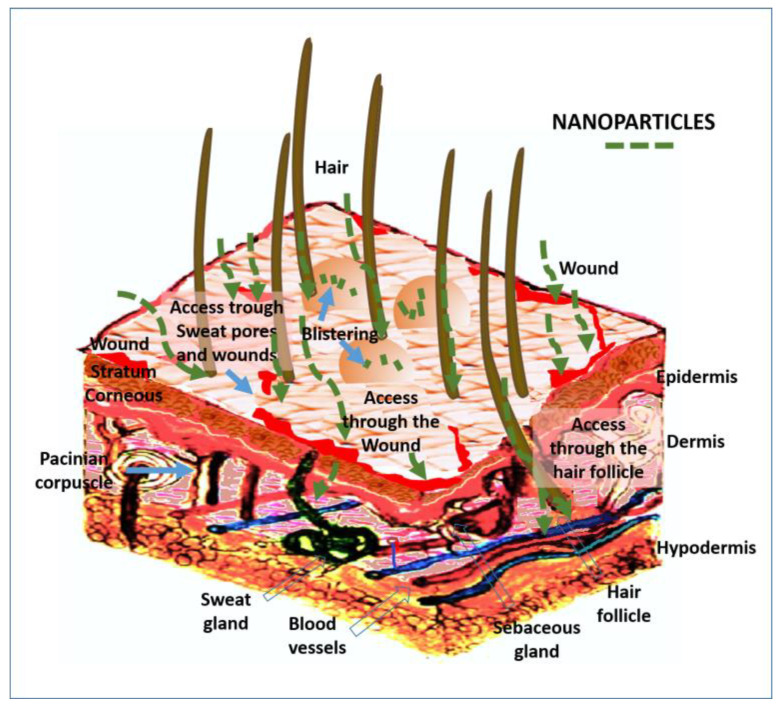
Access of nanomaterials through the pores and wounds of the skin.

The access of nanoparticles through the skin, typically through the pores of hair follicles and wounds, is mainly controlled by the particle size (Figure 9). Thus, particles smaller than 10 nm can enter, causing cell damage, unlike particles larger than 30 nm. Phagocytosis and endocytosis can lead to erythema, edema, and eschar formation [292].

**Table 1:** Access routes of nanomaterials into the human body.

Access Route	Translocation Mechanisms	Affected Organs
Nasal	Inhalation and transfer to the pulmonary region Access to the alveolar epithelium Access to interstitial areas Access to the circulatory system and distribution throughout the body	Nose, pharynx, larynx, trachea, lungs: bronchiolar and alveolar region [270,276]
Access to circulatory and cardiovascular systems	Nanomaterials reach the blood through the alveoli Translocation to the lymphatic system and distribution along the body organs by both systems Translocation to the placental barrier	Hearth, cardio-pulmonary organs, lymphatic system, placentary blood vessels, and fetus body in pregnant women [197].
Access through the olfactory way	Translocation through the olfactory nerves to the brain	Brain [279,280]
Access through the eyes and tear ducts	Access to the cornea and retina Translocation to the central nervous system through the optic nerve	Eyes: Retina and cornea [283] Brain and central nervous system [281,282]
Oral and gastrointestinal region interactions	Access through the mouth Migration from the esophagus to the stomach Translocation to the liver, pancreas and intestines (large and small)	Gastrointestinal organs Stomach, liver, pancreas, large and small intestine [287]
Access to the urinary track	Translocation through the blood to the kidneys Migration to the urinary bladder	Kidneys, urinary bladder [289]
Access through the skin	Internalization through the hair follicles pores and wounds	Epidermis, dermis, sweat gland [292]

## 5. Diagnostic Tools

Particle size determination is one of the most important criteria for determining the degree of the toxicity of nanoparticles. The main techniques used are Dynamic Light Scattering (DLS), Zeta Potential (ZP), Atomic force microscopy (AFM), and Transmission Electron Microscopy (TEM). Each of these techniques has its advantages and disadvantages.

### 5.1. Particle Size Measurement by Optical Methods

The particle size distribution obtained by the DLS technique consists of measuring the fluctuation of light caused by the displacement of particles due to Brownian motion, assuming an optical model based on spherical particles. DLS is useful for measuring isolated, low-polydispersion, highly homogeneous, and non-agglomerated particles in low concentrations [293]. In this sense, ISO 22412:2017 specifies the application of DLS to calculate the hydrodynamic particle size average and the size distribution of mainly submicrometric-sized particles, emulsions, or fine bubbles dispersed in liquids. On the other hand, DLS is also referred to as “quasi-elastic light scattering (QELS)” and “photon correlation spectroscopy (PCS)”, being PCS the technique used to estimate the particle size [294]. However, some experiments report how the requirements of dilution and homogenization procedures for obtaining the DLS measurements strongly affect the aggregation state, producing a higher apparent fraction of individual nanoparticles and underestimating the presence of aggregates inherent to the material [223]. These conditions rule out its usefulness for measuring rectangular-shaped particles, including all those nanomaterials iforming filaments or nanotubes, agglomerated particles, and a high concentration [223]. Even an increased concentration can increase agglomeration; then, the recommended reliable measurements are only at dilute concentrations (typically 50–100 μg/mL) [295]. In most cases, it is unlikely to meet these requirements, even more so in the case of assessing the toxicity of nanomaterials.

The particle size measurement by the zeta potential (ZP) method is carried out by applying an electric field through the sample and calculating the velocity that the particle acquires per unit of the applied electric field by using the laser doppler velocimetry (LDV) technique [296]. The LDV method measures the electrophoretic mobility of the particles by calculating the ratio between their velocity and that of the applied electric field. The advantages of the LDV technique include having a wide dynamic range (10^−4^ to 10^5^ cm/s), not causing any interference, or not being affected by variations in density and temperature. However, one of its disadvantages is the low signal/noise ratio, producing a weak scattered light intensity. In addition, it requires a light wavelength alignment and sometimes seed particles to increase the signal-to-noise ratio, besides being expensive [297]. However, the zeta potential measurement depends on the pH concentration and conductivity of the dispersion medium [298]. The ZP values could become more positive in acidic pH or negative in a basic pH [299]. Furthermore, experts recommend measuring the pH before and after the measurements in different concentrations and considering the dispersion medium’s composition [296]. A study comparing the DLS and ZP methods highlights the advantages and disadvantages. Among the disadvantages, it stands out that neither of the two methods can perform measurements in high concentrations. It is impossible to predict the behavior of NP in blood since they do not function in this medium [295].

### 5.2. Morphological, Chemical, and Structural Properties by Microscopic Techniques

Among the microscopic characterization tools, atomic force microscopy (AFM) and transmission electron microscopy (TEM) techniques are the most recommended.

The atomic force microscope (AFM) can detect forces of the order of nano-newtons. When tracking a sample, it continuously records the topography using a sharp, pyramidal, or conical probe or point, magnifying surface features up to one hundred million times and producing three-dimensional images of the surface. The properties and dimensions of the cantilever and sharp tip play an essential role in determining the resolution of the AFM. Unlike the conventional high vacuum TEM technique, the microscope allows for the characterization of samples in air or fluid environments, making it possible to analyze the diverse type of samples in their native state [300]. This advantage is beneficial in cell characterization [301] since it facilitates the analysis of the cell–nanoparticle interaction. Among its disadvantages, the mounting of samples may be challenging, resulting in difficulty in adjusting the sample perfectly perpendicular to the tip, which causes some tilt that is absent on the sample surface. Other sources of artifacts include thermal drift and non-linearity in the scanner [302].

Transmission electron microscopy in the TEM (Transmission electron Microscopy) and STEM (Scanning Transmission Electron Microscopy) modalities under high vacuum conditions is a tool that allows for characterizing nanoparticles morphologically, chemically, and structurally, directly on each particle. Morphological details allow for differentiating if the particles are agglomerated or aggregated. It is possible to measure their thickness within the agglomerates and identify primary particles that constitute the aggregated particles. The complementary techniques, energy dispersive X-ray spectroscopy (EDS) and energy loss X-ray spectroscopy (EELS), allow for determining the chemical composition. The electron diffraction technique allows for the determination of the crystallographic structure of the compound. In addition, in high-resolution TEM (HRTEM) and high-resolution (HRSTEM) equipment, it is possible to observe the image on an atomic scale. It provides information about the local degree of defects and structural information through the Fourier transform (FFT) technique [303]. However, it is essential to determine the most appropriate voltage conditions to avoid damage by the electron beam due to high voltage effects in highly reactive compounds, producing phase changes, dehydration, or amorphization of the nanomaterial [304]. Another modality of Transmission electron microscopy is Environmental Transmission Electron Microscopy (ETEM), which allows for studying the samples directly exposed to gas or liquid environments [305], and cryoTEM, for studying the nanomaterials’ behavior in low-temperature conditions. These tools provide possibilities for investigating in situ chemical reactions and understanding both the interaction of fast electrons with gas molecules and the effect of the presence of gas on high-resolution imaging [306]. Details of the recent characterization techniques are found in [307].

## 6. Main Applications of Nanomaterials in Protection and Restoration Processes and Risks of Toxicity

The following section describes the existing data on the applications and toxicity of the main nanomaterials used in conservation work on materials, divided, according to their function, into consolidating self-cleaning and biocidal, water-repellent, and hydrophilic (fire-retardant) nanomaterials. Table 2 summarizes the detailed information on its application and the main toxicity risks reported.

### 6.1. Consolidant Nanomaterials

The use of nanostructured hydroxides of different chemical elements has become the primary tool for treating materials in restoration and conservation [308,309]. Among them, calcium hydroxides (Ca(OH)_2_), magnesium hydroxides (Mg(OH)_2_), Calcium phosphate hydroxides, and strontium hydroxides (Sr(OH)_2_) are pretty common [89,310].

#### 6.1.1. Calcium Hydroxide

In the case of the treatment of stone materials, an essential aspect is to define a nano product with the most outstanding compatibility with the substrate [311]. That is why it is recommended to use calcium hydroxides for the consolidation of limestone and marble-type materials [94,312] or magnesium hydroxides for dolostone [1]. In these cases, it is crucial to bear in mind that an unwise choice or an excess of nanomaterial can lead to changes in the aesthetics of the surface due to mineralogical reactions [313] that give rise to reactions such as the dedolomitization of dolostone or dolomitization in limestone [314]. In all cases, the recommendation is to consider the concentration of calcium hydroxide and the concentration and type of solvent used [312,315].

Calcium hydroxide crystallizes on porous surfaces and cracked areas. Due to the effect of the reaction with ambient CO_2_, a carbonation reaction is produced [316,317], leading to phase transformation processes that give rise to different polymorphs of calcium carbonate, which crystallize in pores and fractures, achieving an improvement in the consolidation of calcareous materials [163,318,319]. These calcium carbonates differ in their crystallinity and symmetry depending on the relative humidity conditions, which can be beneficial or lead to adverse effects on their effectiveness as a consolidant. For instance, in low-relative-humidity conditions, carbonation is slowed down or is null, leaving signs of the formation of amorphous carbonate when exposed to humidity close to 50% [320]. With the increase in relative humidity [321], and over time [164], the transformation of calcium hydroxide to calcium carbonate polymorphs, calcite, vaterite, or aragonite is expected. Aragonite crystallizes in highly humid environments, generally associated with the unstable vaterite phase [320]. The more stable phase, calcite, can appear when the humidity is close to 55% [321]. Reported studies show that consolidation differs depending on the crystallinity or the kind of polymorph present [322,323]. For instance, amorphous calcium carbonate studies report how it can significantly improve the surface strength, compressive strength, and weather ability in calcareous materials, with a slight influence on water vapor permeability [324].

Advances in nanostructured calcium hydroxide synthesis techniques seek to obtain nanostructured materials capable of transforming to calcium carbonate in a short time [325,326,327]. At the same time, they manage to be effective traps of environmental CO_2_, and the acceleration of the carbonation process with the formation of the most stable and effective phase, known as calcite, speeds up and improves the effectiveness of nano-compounds [328,329].

However, different morpho-structural properties of nanostructured calcium hydroxide occur depending on the synthesis technique and, hence, the growth of calcium carbonate polymorphs with different morphologies or particle sizes. Among them, aragonite generally crystallizes, developing prismatic or acicular forms that can become harmful in cases of dispersion into the environment. In the same way, an excess of calcium could produce high calcium calcite, which behaves similarly. Apart from its effectiveness as a consolidant, due to its structure and easy carbonation, calcium hydroxide is quite effective in capturing CO_2_ from the atmosphere [330,331].

Another critical issue to resolve is the deterioration produced in wood, canvas, or old paper. Therefore, to solve this problem, it is necessary to modify the pH by deacidifying it by applying calcium hydroxide nanoparticles [332].

Toxicity: Alkali materials, including calcium hydroxide, magnesium hydroxides, and calcium oxides or mixes, can cause dermatitis, severe skin burns [333], or eye injuries [334], also affecting the upper airways [335], the nanostructured calcium hydroxide being slightly more toxic than the larger varieties [335]. Previous studies report how accidental ingestion can damage DNA by generating free radicals and conducing genomic instability through ROS generation that can affect the liver, brain, and bone marrow tissues [336]. The internalization of Ca(OH)_2_ by ingestion can increase the blood pH, which can cause organ damage [335]. Among them, high doses of calcium hydroxide might produce dentinal tissue destruction in that leads to chronic inflammation and necrosis [337]. On the other hand, occupational reports indicate how calcium carbonates that are derived from calcium hydroxide can induce pulmonary diseases in workers [338], even more so when the particle size decreases nanomaterials [339].

#### 6.1.2. Magnesium Hydroxide

Magnesium hydroxide has been proven to be an efficient consolidant in Magnesian rocks such as dolostone, a calcareous rock rich in Mg and Ca [340]. At the same time, reports indicate its high compatibility with the human body and the environment. Studies on the restoration process of old paper apply magnesium hydroxide nanocoatings on cellulose fibers with different refining degrees. The surface modification with Mg(OH)_2_ nanoparticles induces an increase in the pH of the sheets to slightly basic values (around pH 8), facilitates the inter-fiber bonding, and additionally enhances the smoothness of the sheets [341].

Toxicity: In the same way as calcium hydroxide, intense and continuous exposure can cause skin burns and eye injuries [342]. Studies report that it has low toxicity, although its high toxicity turns out to be unstable depending on the local pH conditions. There is a concentration-dependent risk of toxicity in values higher than 625 μg/mL, as observed in specific conditions, which could be dependent on its solubility [343]. The same study using colloidal nanoparticles indicates that the release of OH^−^ or Mg^2+^ ions can induce its toxicity. However, cytotoxic properties can occur, coinciding with the formation of a nanoparticle crust [343].

#### 6.1.3. Calcium Phosphates

Hydroxyapatite (Ca_5_(PO_4_)_3_(OH)) is the main mineral used among the calcium phosphates. It has recently been used in consolidation processes of different materials, mainly in limestone, chalk stone, stucco, and marble or for restoring fossil pieces due to its low solubility compared with calcium hydroxide and calcium carbonate [344], which facilitates the filling of pore spaces and fractures, achieving an improvement in its cohesion [345]. Fluctuations in pH modify the stoichiometry of the crystallized phases [346,347], producing different hydrated phases within the apatite group. which may coexist and crystallize during the consolidation process, depending on the hydration degree of the nanomaterial. Brushite (CaHPO_4_·2(H_2_O) is one of them, and it is frequently identified during consolidation treatments [348]; its crystallization is highly dependent on the pH and the starting Ca/P ratio [348]. In this sense, several consolidation treatments report the hydroxyapatite-brushite coexistence, such as those crystallized during the in situ electrochemical synthesis of sandstone, which appears to fill the pores [345]. Several reports identify the coexistence in calcareous rocks, as observed in limestone after a treatment using poultices [348].

Toxicity: Calcium phosphate has different toxicity than Ca and Mg hydroxides, mainly due to its very low solubility. Its internalization can increase intracellular calcium concentration after endosomal absorption and lysosomal degradation [349]. The tendency of hydrated forms such as hydroxyapatite is to deposit in the urinary system, contributing to the formation of kidney stones. Its interaction with renal epithelial cells leads to oxidative stress due to its entry by endocytosis and accumulation in lysosomes, which produces inflammation and cell necrosis. Furthermore, the inhalation and subsequent entry into the bloodstream during high doses and prolonged exposures can lead to adverse effects [349]. Studies have shown that particles of a smaller size, with a higher specific surface area and a longer aspect ratio, are more toxic [232]. However, the comparison between the effects of various morphologies on toxicity indicates that internalization is more significant in small spheres, followed by needles and rods, with plates being the least toxic. In all cases, toxicity is a combination of the specific surface area, particle size, and aspect ratio as the main criteria [232]. Concerning Brushite, reports identify the increase in nephropathies in patients with nephrolithiasis [350].

##### Calcium Carbonate Phosphates and Other Ion Substitutions

Within the apatite group, in addition to existing hydrated phases, other ion substitutions can occur due to solid solution intrusion processes or substitutional solid solutions [351], the most common being fluor apatite and chloroapatite, with different variants depending on the content of F/OH/Cl ions [351]. On the other hand, substitutions of (PO_4_)_3_^−^ anions by (CO_3_)_2_^−^, (BO_3_)_3_^−^, SiO_4_)_4_^−^, (VO_4_)_3_^−^, and OH by CO_3_ or BO_2_ can occur [352]. In addition, non-stoichiometric Ca/P substitutions are frequent in biomineralization processes, where the calcium content can increase, and, at the same time, carbonate ions appear in high amounts [351,353]. All the possible mineralizations present different symmetries [354] and crystallinity degrees [355] in minerals of the apatite group of a geological type, synthetic minerals, or minerals modified by biological origin [356].

On the other hand, it is possible to replace the Ca cations with other elements in the apatite lattice, such as Ag, Cu, Fe, Zn, Co, Mn, Na, K, Mg, Sr, Pb^2+^, Mn^2+^, or rare earth [357].

Incorporating different elements into the apatite structure makes it possible to obtain nanostructured compounds that are very useful in bone or enamel regeneration, as occurs with the incorporation of Zn ions, turning out to be, in addition to consolidating, effective antibacterial ions [357]. In the same way, the incorporation of silver is promising, although the antibacterial effectiveness can vary depending on whether it is carried out by ion exchange or coprecipitation [357]. In addition, the substitutions also include hydroxyapatite-carbonate structures, typical of samples of biological origin, defined as “carbonated apatites” and classified as type A, where CO_3_ is replaced by (OH)^−^, type B, where the anions (CO_3_)_2_^−^ can replace both (PO_4_)_3_^−^ and (OH)^−^), and type AB, corresponding to a mixture of A and B [357]. For these reasons, the minerals of the apatite group of type B are also quite attractive for heritage conservation treatments, as observed in coatings with consolidating purposes [358]. The incorporation of silver, strontium, barium, potassium, or zinc to the type B compound demonstrated good adherence and an improvement in the mechanical resistance of the stone material in freeze–thaw tests [358].

Toxicity: Similar to the patite hydrated phases, there is a greater risk of stone formation with these compounds in the kidney [359,360]. This area has a higher risk due to the accumulation of highly carbonated apatites [357,361]. However, one of the main topics of debate so far is to determine to what extent the carbonation level of apatites could be one of the factors responsible for urinary tract infections [362]. However, it is necessary to consider that carbonate and bicarbonate ions are present in physiological fluids [362], which come from membrane vesicles secreted by various cells, including vesicle matrices (a type of extracellular vesicle) [359]. Specifically, vesicle matrices, due to their nanometric size, turn out to be mineralization nucleation agents due to an increase in the concentration of calcium and phosphates [359], leading to ectopic calcifications, with a greater probability of an increase in arthritis processes and arteriosclerosis in addition to kidney disease or hyperlipidemia [359].

On the other hand, there are toxicity risks when incorporating different elements in the mineral series of the apatite group. Many of them correspond to trace elements, which exert their effect in transforming the mineralogical phases, being able to modify the morphology of the crystals [363]. Among the most studied in terms of toxicity are zinc, iron, and strontium, in which pathological calcifications have been identified [363]. Specifically, the studies on Zn in the apatite network of biological origin indicate an association with osteoarthritis, osteoporosis, and kidney stones [363]. However, determining whether or not Zn occupies the apatite lattice is still under debate. However, fundamental advances using crystallography have helped to clarify this dilemma [363]. Specifically, pathological calcifications of apatite studied by X-ray absorption revealed that Zn did not occupy the apatite network but appeared outside [363]. Finally, the authors pointed out that this location could affect details such as crystalline morphology and its degree of toxicity, which could be a key parameter in pathological calcifications of oligo-elements [363].

### 6.2. Protective Treatments Using Hydrophobic Coatings

#### Amorphous Nanosilica

One of the most widely used nanomaterials in surface protection processes against water action is SiO_2_ in its amorphous variety with a short-range order structure. Its hydrophobic properties demonstrate high efficiency by depositing layers of amorphous nanosilica on substrates exposed to the action of aqueous substances [10,18]. In wood treatments, it can reduce swelling and helps to prolong the wooden structure. Due to its specific properties, several studies report its use for fire resistance, self-cleaning, and scratch resistance without producing aesthetic changes in the material [1].

Toxicity: Amorphous silica nanoparticles can induce cytotoxicity and genetic or epigenetic alterations in humans due to the impact of the nano silica/bio-systems interface on the cellular and biochemical processes [364]. During the conservation processes, there is a risk due to inhalation that can generate inflammatory processes in the lung submucosal cells in specific doses and particle sizes around 10 nm. Around this particle size, the induced ROS leads to apoptosis, decreasing cell survival SiO_2_ [364,365]. Moreover, as observed in spherical particles, its penetration, translocation and deposition are all affected by their properties, such as the size, shape, or surface properties [219]. Some studies emphasize that its toxicity depends mainly on its specific surface rather than the aggregation degree [366].

### 6.3. Self-Cleaning and Biocides

Biological action is one of the most outstanding deterioration agents, affecting buildings, mural paintings, canvases, papers, or archaeological pieces. The interaction of fungi and bacteria with the substrate leads to textural and compositional modifications of the material that finally cause its decay. The most commonly used antibacterial and antifungal agents are TiO_2_, ZnO, Ag, and MgO, which, in their nanometric version, achieve successful results [367]. In the case of metal nanoparticle applications with microbial properties, statistical analyses report that silver is the most studied and reported metal (63%), followed by copper (9%), zinc (9%), gold (8%), iron (6%), magnesium (3%), platinum (1%), titanium (1%), and vanadium (1%) [368]. Partly, the economic cost associated with gold and platinum limits their synthesis and application [368].

#### 6.3.1. Titanium Dioxide

Titanium dioxide is a photocatalyst widely used as nanocoating for controlling biodeterioration and serving as a self-cleaning agent against pollution [369,370]. Among the different isomorphs of TiO_2_, the anatase phase displays the highest photocatalytic performance [27]. A surface treated with titanium dioxide can keep clean by the action of sunlight and rainwater based on its photocatalytic and super hydrophilic properties [371]. At the same time, TiO_2_ can become self-disinfecting against pollutants that break the contaminants into water and CO_2_ in the presence of light. Gradually, the adsorbed organic dirt decomposes by TiO_2_’s photocatalytic property, while organic contaminants and dust are removed with rainwater due to TiO_2_’s superhydrophilic properties. However, its effectiveness depends on the lighting conditions, the amount of rain, or the dirt accumulation rate [371].

The photocatalysis process causes oxidizing reagents, which conduct the decomposition of many organic substances that deposit on or form on surfaces [372]. The biocide action occurs when a treated surface with the anatase phase of titanium dioxide is exposed to UV light and responds by accelerating the oxidation process and eliminating organic matter such as fungi and algae [373].

On the other hand, to improve the photocatalytic performance, the photoelectrochemical technique, a fusion between electrochemistry and photocatalysis, improves the antibacterial efficiency, as observed in TiO_2_ nanowires, whose antibacterial efficiency is ten times higher than that of the traditional photocatalytic method [374].

Toxicity: The main risk in conservation processes occurs during materials management. Reports indicate that the generation of oxidative stress caused by internalized nanoparticles into the mammalian cell produces inflammation, cytotoxicity, damage in the DNA [375], and carcinogenic effects by inhalation [48]. The structure of TiO_2_ polymorphs plays a role in its toxicity degree, so many studies report that the anatase phase is more toxic than rutile [376]. Comparing both phases under UV light excitation, the most inactive catalytic materials (rutile) could be less cytotoxic than similarly sized anatase nanoparticles. In addition, in any case, it is essential to consider that the higher the toxicity, the greater the ability to generate reactive oxygen species under ultraviolet lighting [251,376]. Furthermore, the particle size could play an important role such that bigger particles behave differently, and their toxicity could be higher in the rutile phase compared with the anatase phase in particles around 200 nm when they are in the absence of photoactivation [251]. Specific studies talk about eventual access by the oral way, and the continued ingestion of TIO_2_ could have the same risk as that observed in food-grade TiO_2_ by maternofetal transfer in pregnant women, which is a reason why the authors recommend the need for the risk assessment of chronic exposure to TiO_2_-NPs during pregnancy [377]. Other studies report the risk of pulmonary damage during work in indoor paints, including nanoTiO_2_, by the eventual release of TiO_2_ nanoparticles powder, conducting inflammation and DNA damage [378]. Furthermore, many works of art have white pigments based on TiO_2_ in oil paintings, which could be a risk during restoration processes [379,380].

#### 6.3.2. Zinc Oxide

One of the main properties of Nano Zn oxide is controlling the biodeterioration processes by inhibiting microbial-fungal attacks and forming a protective surface layer to enhance its durability due to its photocatalyst properties [381]. However, as in all cases, its effectiveness depends on specific physic-chemical characteristics. For this reason, researchers frequently focus on improving them by changing the synthesis conditions [107,382].

Toxicity: The primary use of ZnO is as a sunscreen for the skin. However, studies indicate that it can be located in the first superficial layers of the stratum corneum [383], increasing its penetrability by wounds and burners’ skin or accumulating in the hair follicle [384]. Although prolonged exposure to ZnO is not frequent in material conservation procedures, experiments carried out with sunscreen report how the release of Zn ions from ZnO could reach the blood [385]. The route of entry of the biocide through the skin can be risky without adequate protective equipment. Its toxicity increases depending on the nanomaterial’s dose, the type of solvent, the concentration, and the specific properties. Furthermore, several studies talk about the risks after internalization including neurotoxicity [386], hepatic and embryonic kidney cells cytotoxicity, oxidative stress, and genotoxicity [387].

#### 6.3.3. Silver

Nowadays, the applications of silver nanoparticles include sectors as diverse as medicine, [388], diagnostic bio-sensors [389], the pharmaceutical industry [390,391] environmental remediation [392], electronics [393], agriculture [394,395,396], forestry [397], food packaging [398,399], and the automotive industry [400]. In the field of heritage conservation, silver is a highly employed nanomaterial against the biodeterioration affecting different materials of cultural heritage [12,401], such as its use in limestones [402], sandstones [403], artworks [404], or textile pieces [405].

Toxicity: Despite its significant advantages, the risk of toxicity in humans, living beings, and the ecosystem comes from multiple sectors such as mining, industry, transportation, or wastewater treatment. Restoration work does not escape this risk, since these nanomaterials are deposited on different surfaces that contribute to dispersing silver in various scenarios affecting the environment when exposed to air or water [406]. For instance, there is a risk of toxicity from the oral way during restoration activities. It is crucial to consider that, among the mechanisms of internalization in the human body of silver nanoparticles, there is ingestion which may conduce to diabetes, hyperlipidemia, or hypertension [289,407,408], medical treatments being an essential way of entry into the organism. Studies in cultured cells and animal tissues reported induced oxidative stress, genotoxicity [409], and apoptosis [410]. Its incorporation produces cytotoxicity inside the mitochondria and nucleus, implicating the direct involvement in mitochondrial toxicity and DNA damage [407]. However, it depends on the specific properties of the nanoparticles, including their size, surface area, shape, agglomeration status, and dose. Their interaction with the cell differs depending on parameters such as diffusion, gravitation, and convection forces [407].

#### 6.3.4. Gold

Gold nanoparticles have shown high efficiency and multifunctionality [411], highlighting their antifungal and antibacterial properties [412]. Treatments using gold nanoparticles to prevent dust deposits on cultural heritage building surfaces demonstrate an improvement in the photocatalytic capacity of TiO_2_, conferring a constant self-cleaning activity through the TiO_2_/SiO_2_/Au mixture [413]. In addition, in wood consolidation processes, Au nanoparticles show high effectiveness when combined with nano-hydroxyapatite (HAP), as reported in the Au/HAP mixture obtained by sonochemical homogeneous precipitation synthesis [414]. Specifically, the Au/HAP nanoparticles could cover vessels and wood fibers and fill empty spaces, thus stopping the weathering process more accentuated in aged wood than in young species [414].

Toxicity: Despite the multiple applications of gold nanoparticles, there is also a risk of toxicity that is still a matter of debate, since some articles maintain that they are not toxic and others maintain the opposite [415,416]. As Sany et al. point out [416], the conflict in the gold bioactivity data is partly due to laboratory protocol variations, making it difficult to determine and generalize vital aspects of its effects without establishing a consensus that allows us to conclude its toxicity effects. However, due to this situation, researchers comment on the need to systematize data on the most relevant physical-chemical parameters that govern and control the toxicity of gold nanoparticles at the cellular level and throughout the organism [415].

On the other hand, specific studies indicate, to a certain extent, oxidative stress in tissues and cell lines used in vivo and in vitro, respectively, with the liver, spleen, and kidney being the most affected [416]. As summarized by Sany et al. in their review, several in vivo experiments in humans report leukemia, lung fibroblasts, or spermatozoa modifications affecting their viability and motility [416].

#### 6.3.5. Platinum

Among the advantages of platinum nanoparticles, their antimicrobial, antifungal, antioxidant, antidiabetic, and anticancer properties stand out [417]. Among them, antifungal and antibacterial properties are strategic for controlling colonization on stone substrates [418]. The studies reported by Khan et al. 2021 [417] provide information about the state of the art concerning the different factors that affect catalytic efficiency, making the nanoparticles’ morphology a crucial factor [417]. For example, an assessment of the efficiency of platinum nanoparticles comparing nanoclusters, nanospheres, and nanocubes indicated that the nanoclusters were more efficient among the three types of morphologies because the high percentage of corners and edges of atoms increased the efficiency of the nanoclusters’ catalytic phenomenon [419]. On the other hand, to achieve greater antimicrobial efficacy, one of the possibilities is to use photochemical sterilization, previously mentioned in TiO_2_ treatments [374]. By means of this technique, it is possible to eliminate micro bacteria photoelectrochemically by applying semiconductor powders of platinum/titanium oxide, which, when irradiated with a xenon laser lamp, inhibit the respiration of microbial cells, as reported in the control of Lactobacillus acidophilus, Saccharomyces cerevisiae, and Escherichia coli [420]. Another possibility is to apply Pt/MWCNT treatments, where it is possible to obtain better results by combining the benefits of carbon nanotubes (described in the next section) with those of platinum, with antimicrobial activity [1]. Furthermore, there is the possibility of making different combinations, such as platinum/silver, whose combination has been proven to be effective in dental treatments for controlling bacterial activity [421].

Toxicity: The assessment of platinum nanotoxicity is a subject on which the different research groups have yet to agree, as reported by Czubacka et al., 2019 [422]. Although the internalization of nanoparticles can occur through the respiratory or digestive routes, there are no studies regarding penetration through the skin [422]. Based on the available toxicity information, there is evidence that nanoparticles can accumulate in the liver, heart, lungs, or kidneys. Still, their toxicokinetics depend on the size of the particles [422]. In addition, platinum nanoparticles can cause inflammation and oxidative stress when they enter the body orally. In addition, intravenous access can cause hepatotoxicity, nephrotoxicity, DNA damage, and cell apoptosis [422]. However, despite the advances in toxicity assessment, more tests are needed to determine the limit values of the occupational exposure of workers [422].

#### 6.3.6. Copper

Copper nanoparticles stand out for their biocidal and antibacterial power, both in the medical sector [423] and in conservation treatments for pieces of cultural heritage [1,15,16]. Specifically, investigations into its application against fungi and lichens in the conservation of archaeological material on marble, sandstone, and plaster substrates indicate a high efficiency during three years compared to traditional treatments [424]. However, its efficiency may vary depending on the climatic conditions to which they are subjected, with the possibility of a slight darkening in color in materials treated with a high concentration of Cu [424]. Another alternative for eliminating biological colonization in art stone works is the mixture of copper nanoparticles with compounds with hydrophobic and consolidating properties prepared from oligomers of ethyl silicate and polysiloxane [425]. Likewise, studies on wood to control termite colonization indicate a high efficiency of copper nanoparticles compared to Zn nanoparticles or micrometric Cu particles [426].

Toxicity: The nanotoxicity of copper nanoparticles is a subject of debate since different researchers maintain that there are many variables to consider, including the effect of size, the oxidation, or the corona effect [423]. Some studies identify a relationship with increased neurodegenerative disorders, including Alzheimer’s and Parkinson’s [427]. At an ecological level, cover nanoparticles are highly polluting and seriously affect tiny aquatic organisms [427].

### 6.4. Multifunctional Properties of Carbon Compounds (Nanotubes, Nanowires, Nanorods)

Today, the use of allotropic nanostructured carbon compounds, classified as fullerenes (zero-dimensional), nanotubes (one-dimensional), and graphene (two-dimensional), has achieved significant advances in different applications, such as in the pharmaceutical, electronics, and biomedicine fields [428].

Carbon nanotubes consist of tubular carbon molecules arranged in monoatomic layers of carbon-forming cylinders, which can be isolated forming monolayers (SWCNT) or concentric arrangements forming multilayers (MWCNT). Depending on the synthesis process, they can develop different morphologies and include other chemical compounds in their structure to achieve certain specific properties. As reported along the evolution of manufacturing conditions, the morphologic variations include bundles, entangled tubular objects, and fibers. Specific bundles may develop crystal-like structures, as reported in hexagonally arranged SWCNT [429]. Moreover, regular or irregular agglomerates can assemble, forming other morphologies such as double spiral rounded, tubular, or flower- bundles shapes [430]. In all cases, the thickness diameters range around 2–3 nm or less, and their length reaches the micrometric order [170,431].

Heritage conservation takes advantage of its versatility for creating new products with specific properties, such as super-hydrophobic or hydrophilic materials [1], to protect from water [432], biocidal [433], or fire action [118,434]. Its use in building materials restoration processes mainly focuses on reinforcement to improve the mechanical strength of SWCNT/MWCNT grown on the polymeric matrix [435], as described below. Moreover, the advantages of fine art conservation materials report the design of devices, including CNT, in the form of lightweight, flexible, transparent, and breathable film-like mats applicable to preserving the required humidification properties and as gas permeable membranes of paintings, textiles, and works on paper [1].

Toxicity: Based on the multitude of variables that can form in both SWCNTs and MWCNTs, it is expected that the level of toxicity will be very different, depending on their morphology, degree of agglomeration, aspect ratio, rigidity and flexibility and the presence of impurities or metal additions (Fe, Co, Ni,), besides the local environment in which they interact. Internalization can be different, obstructing critical blood pathways or producing various cytotoxic effects [436]. In all cases, the degree of damage will depend on the dose and the time of incubation [437]. The smaller the diameter of CNTs and the greater the aspect ratio, the greater the toxic effect that is observed [438]. Increasingly, reports with different connotations discuss how the interaction of CNT induces damage to the heart and cell proliferation, muscles, hindrance in the blood flow, vascular atherosclerosis or in the alveolar and intra-tracheal tissue walls [439]. Moreover, CNTs accumulation in the kidney or the liver may lead to renal and liver toxicity [439]. In other cases, they can induce lung fibrosis [440], ocular damage [441], or granuloma formation with entrapped MWCNT agglomeration in the subcutaneous tissues [439]. Reports indicate how CNT can produce inflammation, apoptosis, and oxidative stress in the brain [442]. Furthermore, specific CNTs such as those with elongated fiber shapes constitute a relatively high risk of producing DNA damage due to mutations, oxidative stress, or chromosome alterations depending on the concentration and specific particle properties [443]. With the advance of research, new studies suggest the necessity of alternative designs and safety application methods for minimizing their damage [443].

### 6.5. Fire Retardants

The protection of different materials against eventual exposure to fire is an issue for which nanotechnology can be helpful. The treatments consist of coatings with products that resist high temperatures or react by decomposing to confer protection against heating [372,444].

Among the nanomaterials with fire-retardant properties [445], magnesium hydroxide [446], aluminum hydroxide [176], and new combinations, such as adding magnesium hydroxides to carbon nanotubes, stand out [118]. Other fire retardants consist of polymers such as phosphines, phosphonates, phosphates, silanes, siloxanes, boric acid, borates, carborane, or melamine derivatives [447], which are highly harmful depending on the exposure dose due to the toxic gas emission with the increase in heating [448]. Furthermore, it is essential to consider a series of factors that determine the toxic potential of materials, including the elemental and organic composition of the decomposed material, based on their thermal properties, and the possible reactions among them [448].

#### 6.5.1. Magnesium Hydroxide–CNT Combinations

Magnesium hydroxide is the most commonly used fire retardant as a protective coating. It hydrolyzes to obtain crystalline water, absorbing a large amount of heat (44.8 kJ/mol) when the temperature reaches between 340 °C and 490 °C [444]. During hydrolyzation, it is transformed into magnesium oxide, releasing water [444]. On the other hand, CNTs are light and flexible, as reported in coating film trials, obtaining promising results [449]. Therefore, the combination of both compounds manages to obtain fire-retardant properties [118].

Toxicity: In addition to the most relevant aspects of the toxicity of both magnesium hydroxide and carbon nanotubes, described in the previous section, specific studies on magnesium hydroxide emphasize the possible risk of Mg^2+^ ion release during heating, which could be higher in high Mg concentrations than in low ones [343]. Moreover, with a high dilution of nanoparticles, a low content of Mg emission happens [343]. Another factor could be the possibility of magnesium hydroxide nanoparticles aggregation, which could generate a thicker layer of material, preventing the release of Mg^2+^ and the stability of the colloid regarding its cytotoxicity. Similarly, it is essential to consider the specific physicochemical properties of carbon nanotubes. Therefore, knowing these properties would be the only way to determine the degree of toxicity of the CNT-Mg hydroxide combination, which has been unreported to date.

#### 6.5.2. Nano Aluminum Hydroxide

Nano aluminum hydroxide stands out for its high efficiency as a fire retardant and consolidant, which decomposes when heated at 320 °C, losing its crystal water [450]. There is an interest in improving its efficiency by preparing composites, including aluminum hydroxide nanoparticles. For instance, as assessed in wood pieces, the mix of foam composites and nano aluminum hydroxide-foaming agents results in a good fire retardant for preventing spontaneous coal combustion, obtaining, at the same time, smoke suppression [450].

Toxicity: Studies about the toxicity of aluminum hydroxide advise how its ingestion can conduce to potential reproductivity in embryo/fetal toxicity [451]. When it is in nanometric size, the risk is higher. The internalization of aluminum hydroxide nanoparticles can produce allergic reactions, besides dermal damage producing erythema, subcutaneous nodules, contact hypersensitivity, and granuloma, as observed during vaccine inoculation [452]. These risks can also occur when handling solutions during the fire-retardant coatings procedures when aluminum hydroxide nanoparticles are applied to the materials without adequate protection, causing skin irritation, which increases with a higher concentration or decreasing particle size.

#### 6.5.3. Nanoclays

Other compounds used as fire retarders are nanoclays. The reported phyllosilicates include montmorillonites and sepiolite nanorods. The ensemble of these clays with polymer chains forms nanocomposites [176]. In the case of sepiolite, a magnesium silicate-containing nanoclay mineral, silica tetrahedra form nanoplatelets surrounded by nanosheets of magnesium. In this case, sinalol groups (SI-OH) give it hydrophilicity and suitability in chemical functionalization [176].

Toxicity: Reports about sepiolite toxicity highlight the high risk of persistent pulmonary inflammatory and cytotoxic effects in humans and animals [453], which could have the same lung toxicity as other nanofibers or nanorods, as described in the previous section. However, its risk depends on the inhaled nanofibers accumulation.

Montmorillonite has the advantage of being an oral delivery carrier because it provides mucoadhesive properties across the gastrointestinal barrier [454]. Reports about its toxicity in in vivo and in vitro studies point out that montmorillonite could cause some cytotoxic effects at high concentrations after long-term exposure [454]. In addition, reports about the incorporation of this clay into polymeric systems talk about intestinal damage producing morphological alterations in the Golgi apparatus and nucleolar segregation due to the increase in intracellular reactive oxygen species production [455].

### 6.6. Hybrid Nanomaterials and Nanocomposites

Nowadays, protective treatments for stony materials use composites based on polymers with nanomaterials [176,456], which vary in effectiveness depending on the type of polymer used. For instance, the new trend is to synthesize organic–inorganic hybrid nanocomposites using acrylate, organosilane (TEOS), fluorinated silane (FOTCS), and titania nanoparticles (TiO_2_) with hydrophobicity and self-cleaning properties, resulting in an opportunity to obtain better thermal, mechanical, and weathering resistance of carbonate stones [457].

Moreover, the mixture of siloxane (hydrophobic) with nano SiO_2_ (superhydrophobic) composites shows that this combination can only slightly reduce the water vapor permeability and the water amounts absorbed by capillarity in comparison with the same polymer without nano SiO_2_. The aggravating factor is that it can produce a modification in color on the surface [124]. Nevertheless, another kind of polymer can produce better results, such as applying Fluor-alkyl silanes-SiO_2_ composites in granites in the appropriate proportion [458]. In the same way, the application of new compounds, including nano TiO_2_ in combination with Fluor-polymers, can enhance their biocide and self-cleaning properties [456]. However, its effectiveness depends on the concentration. Among the disadvantages, there is a risk of producing significant damage in the treated stone by the titanium oxide–polymer interaction [456]. Some reports explain how coupling the photocatalytic titania with the hydrophobic polymer could lead to low contents of water-soluble ions adsorbed by the NPS, which may be accumulated on the coated stone surface [456,459]. Within the protective treatments using polymers, the most recent line of research opts for incorporating SWCNT and MWCNT to improve the materials’ mechanical and UV radiation resistance. Single- and multi-walled CNTs are promising superhydrophobic materials in combination with milled xerogel or poly (dimethylsiloxane), causing a decrease in the water adsorption capacity of the polymeric matrix [153].

Furthermore, new combinations seek to improve the biocide action, such as citrate-stabilized silver nanoparticles, silver/TiO_2_ nanocomposites, and citrate-stabilized silver/TiO_2_ nanocomposites, whose effectiveness depends on their penetration depth in the stone matrix [103]. Furthermore, combinations such as Silver-TiO_2_-SiO_2_ have improved photocatalyst properties for self-cleaning applications [460], along with SiO_2_ crystalline-TiO_2_ nanocomposites, which produce photoactive and hydrophobic coatings when applied as sols brushed onto stone [461].

Toxicity: The toxicity risks of polymers with nanoparticles are an issue that needs more investigation. It is a priority to analyze the possible risk of these composites since the polymers are easily degradable by the action of UV rays, exposing the layer of nanomaterials on the surface to which it was applied, as reported in MWCNT [462,463], with the possibility of environmental dispersion [464], depending on their degree of adherence. Therefore, it is necessary to consider the emission risk during handling procedures such as cutting, dry coating, or grinding that will contribute to eventual toxicity. [465].

In addition, there is also a risk of environmental dispersion in other types of exposures, such as that produced by the sun (photodegradation), thermal variations (thermal decomposition), humidity (hydrolysis), interactions with other solutions (chemical processes), or incineration [465]. Therefore, it is necessary to delve into this topic since, for instance, a change in the material’s roughness or particle size is not exempt from damage due to the mentioned factors, which facilitate its degradation and the consequent release of the nanomaterial.

Therefore, the exposition of environmental conditions, including the wind speed, the presence of chemically enriched water, the relative humidity, or the specific climatic conditions of the different types of nanocomposites, could have the same risks as nanoparticle powders in the short and long term depending on the particular properties of the composite.

The constant increase in the hazards of nanomaterials during their handling tasks, or during grinding, cutting, or shredding processes, has encouraged different specialists in nanomaterial synthesis to promote the substitution of high-risk nanomaterials for less dangerous ones.

Although most of the trends are focused on improving the effectiveness of nanomaterials in their different applications, it is crucial to consider that their toxicity appears right from the moment of synthesis in which precursors are used that can affect the ecosystem during their manipulation, including disposable material that can leak by water or air. For these reasons, there is currently a tendency to develop new nanomaterials through green synthesis and bioinspired nanomaterials [466].

The objective of the green synthesis technique is to produce nanomaterials by achieving a biogenic reduction in metal precursors. Its advantages include being ecological, low-cost, and free of chemical contaminants, using bottom-up techniques [467]. Specific bioinspired nanomaterials promote the manufacture of different compounds such as metal hydroxides/oxide, silver, titanium oxide, zinc oxide, and nickel oxide nanoparticles using natural resources, which have significant use in restoration processes of cultural heritage [466,468,469,470].

Among them, compilation reports mention the diverse green synthesis method and its potential applications [250] for obtaining biogenic silver by mixed-valence polyoxometalates or polysaccharide methods [471] or by including extracts of coffee and tea [472]. Similarly, other applications include the use of aloe vera as a precursor to obtaining silver [473], a mix of lemon juice, zinc acetate, and sucrose for fabricating nano ZnO [474], or different extracts for obtaining TiO_2_ nanoparticles [475].

Although the new trend among groups specialized in synthesis processes allows for a decrease in the impact of the different reagents on the environment, there is still much to study about their toxicity.

Once released and airborne, it is necessary to evaluate its specific properties, the different aggressive factors with living beings, and the environmental media. These advances in knowledge are the only way to know the degree of toxicity of each of the nanomaterials obtained by green synthesis methods and the different bioinspired options.

**Table 2:** Main nanomaterials and new alternatives used in conservation treatments of cultural heritage and the risk of toxicity.

	Product	Properties	Reported Toxicity
Consolidants (artworks, calcareous materials, mortars)	Ca(OH)_2_	Stone: [1,15,16,91,94,95,96,323] Artworks [4]	Dermatitis, skin burns [333], eye injuries [334], DNA damage [336], Lung diseases [338].
	Mg(OH)_2_	[444]	Skin burns and eye injuries [342]
	Mg(OH)_2_ CNT	[118]	Not reported
Stony materials consolidants	Hydroxiapatite Brushite Calcium carbonate phosphates (with or without metallic derivatives)	[348] [358] [357,358]	Kidney stones, nephroliiasis [359] Ectopic calcifications and an increase in arthritis and arteriosclerosis [359] Kidney stones, hyperlipidemia [359]
Hydrophobic/consolidant	Amorphous SiO_2_	[1,10,12,15,16,18,153]	Inflammatory processes in the lung submucosal cells [364,365,366]
Biocides: self cleaning	TiO_2_	[369,371,372]	DNA damage, lung diseases, carcinogenic by inhalation, fetus damage [45,48,49,50,378,379]
	Zn oxide	[107,381,382]	Neurotoxicity [386], hepatic/embryonic cytotoxicity, genotoxicity [387]
Biocides: self cleaning	Silver	[12,401,402,403,404,405]	Diabetes, hyperlipidemia, hypertension [289,407,408]
	Gold	[411,412]	Oxidative stress in the liver, leukemia, lung fibroblasts, or spermatozoa modifications [416]
	TiO_2_-SiO_2_-Au	[413]	Not reported
	Au-HAP	[414]	Not reported
	Platinum	[417,418]	Hepatotoxicity, nephrotoxicity, DNA damage [422].
	Pt/MWCNT	[1]	Not reported
	Platinum/silver	[421]	Not reported
	Copper	[1,15,16,424,425,426]	Neurodegenerative disorders [427]
Hydrophobic, antimicrobial, consolidant strengthener	Carbon compounds (nanotubes, nanowires, nanorods)	Super-hydrophobic [1] Mechanical properties strengthener [1] Gas permeable membranes [1]	Atherosclerosis, blood alteration [439] Heart, alveolar, and intra-tracheal damage [439], Renal and liver damage [439] Inflammation, apoptosis, and oxidative stress in the brain [442] DNA damage, oxidative stress, or chromosome alterations [443]
Hydrophilic	Magnesium hydroxide	[1,2,13,15,16]	Skin burns and eye injuries [342]
	Mg(OH)_2_/CNT	[118,449]	Not reported
	Aluminum hydroxide	[176,450]	Embryo/fetal toxicity [451], dermal damage [452]
	Nanoclays with polymers	[176,450]	Pulmonar inflammation [453]
	Montmorillonite	[176]	Cytotoxicity [454], intestinal damage [455]
Protective treatments: hydrophobicity and self-cleaning	Hybrid nanomaterials and nanocomposites	[8,176,456,457,458]	Ecosystem damage [465]
		TEOS/FOTCS/TiO_2_ [457]	Not reported
		Silver/TiO_2_ nanocomposites [105].	Not reported
		Silver/TiO_2_/SiO_2_ [460]	Not reported
		Citrate-stabilized silver/TiO_2_ nanocomposites [103].	Not reported

## 7. Role of International Organizations in the Control of Exposure to Nanomaterials and the Assessment of the Degree of Toxicity

Over time, the constant progress in nanotoxicity cases has led different sectors, mainly nanomaterials factories, to consider how to identify hidden risks that could affect workers.

After knowing the primary published data on nanomaterials typically used in heritage conservation and the possible toxicity risks, it is crucial to question what organizations have done in this regard. The great concern and constant increase led different groups to create guides for workers, which were compiled by the European Commission [198], based on individual European states regulations, and other more recent ones that were incorporated including:

- Austria (Nano information) [476,477]
- Danish Environmental Protection Agency Denmark (NANORISKCAT NRC) [478]
- France (French Agency for Food, Environmental, and Occupational Health & Safety ANSES 2008-INRS) [479]
- Germany Bundesanstalt für Arbeitsschutz und Arbeitsmedizin BAUA, German institute for Standardization -DIN eV, -Federal Institute for Materials Research and Testing (BAM)) [480]
- Italy (INAIL 2011), Italian National Institute for Occupational Safety and Prevention, Department of Occupational Medicine Italy [481]
- The Netherlands: Health Council of the Netherlands and Delft University of Technology (TU Delft): Nanosafety Guidelines [482,483]
- Switzerland Bundesamt für Gesundheit (BAG) (INFONANO), nanotechnology [484]
- Spain (Spanish Health and Safety Institute (INSST)) [485,486]
- UK (Health and Safety Executive (HSE) and the British Standard Institution (BSI)) [487,488]

Some of the decisions of the European Commission were to establish manuals and guides for workers, to reach a consensus within the different groups, and to commit to updating them periodically [489,490,491,492].

On the other hand, the United States, by means of the National Institute for Occupational Safety and Health (NIOSH), which stands out for having a solid support group for nanotoxicology, constituted the NIOSH Nanotechnology Research Center (NTRC) in 2004, which frequently reports the last advances in the field of occupational health and safety [493,494]. The United Kingdom, through the British Standard Institution (BSI), has also established its quantitative evaluation database considering its solubility, carcinogenicity, mutagenicity, estrogenicity, or toxicity for reproduction [487,488]. Their committee and different research groups frequently update their advances [495].

Other organizations represent countries such as Canada (Occupational Health and Safety Research, IRSST) [496], Australia (NanoSafe Australia) [497], or China [498]. This information and compilations of global safety process standards are available in review articles [499,500].

The following considerations were established by the European Comission [198]:

In the first place, the premise focused on establishing a definition of what a nanomaterial is, defining it as a particle that has one or more external dimensions within a size range of 1–100 nm. As a priority, it was necessary to establish the most appropriate evaluation process and the way to manage risks.

The following steps were defined. The first one was the identification of nanomaterials in the workplace, giving information on constituents, mixtures, and physical-chemical properties. The second step would be the evaluation of the risks for workers due to inhalation, absorption through the skin and eyes, ingestion, fires or explosions, chemical reactions, risks derived from the facilities due to leaks or spills, protection trays against mechanical impact, or the existence or inexistence of preventive maintenance. The establishment of these categories would be dependent on the degree of concern, among which it is necessary to consider the shape (nanoparticle, nanofiber, nanoplate, nanorod, nanotube, or nanowire), solubility in water (high, medium, or low), and biopersistence (ability to resist removal by natural mechanisms). Similarly, it is necessary to determine the amount of dust, its probable emission, duration, and frequency, and the number of exposed workers.

One of the significant advances in the control of the Environment, Health, and Public Safety is the constitution of an intergovernmental Organization for Economic Cooperation and Development (OECD), where representatives of 36 industrialized countries of North and South America, Europe, and the Asia-Pacific region meet, as well as the European Commission, to coordinate and harmonize policies, discuss issues of mutual interest, and work together to respond to international issues such as essays and evaluation; good laboratory practices and compliance monitoring; pesticides; biocides; risk management; the harmonization of regulatory oversight in biotechnology; the safety of novel foods and feeds; chemical accidents; pollutant release and transfer registers; emission scenario documents; the safety of manufactured nanomaterials; and pathways of adverse outcomes [501]. OECD was one of the first major international treaty organizations to establish nanotechnology groups. In 2006, OECD’s Council established the Working Party on Manufactured Nanomaterials (WPMN) as a subsidiary body of OECD’s Chemicals Committee [502]. WPMN, also known as WPN, has the purpose of advising on emerging policy issues in science, technology, and innovation related to the responsible development and use of nanotechnology [502]. Details about the constitution of the different organizations and standardization criteria up to 2011 are available in the Nanotechnology Standards book [503].

OECD provides free-access information on the safety of manufactured nanomaterials [504], starting its first report in 2006 [505]. Between the years 2006 and 2015, OED established specific details such as the safety of manufactured nanomaterials, methods and models available for assessing exposure to manufactured nanomaterials, testing guidelines on the ecotoxicology and environmental fate of manufactured nanomaterials, inhalation toxicity, genotoxicity, and guidance manuals for integrating risk assessment in the life cycle assessment of nanotechnology-enabled applications to finally establish preliminary guidance notes on nanomaterials, detailing interspecies variability factors in human health risk [504]. Once OECD established the main parameters, the new focus was the specific considerations, such as using dissolution as a function of surface chemistry to assess the environmental performance of nanomaterials in risk assessments using silver nanoparticles [506].

The 2016 year stands out by the fundamental advances in critical considerations such as physical-chemical parameters, relevant measures, and methods for regulating nanomaterials. In addition, it includes the categorization of nanostructured materials, updates in the delegations on the safety of manufactured nanomaterials, and compilations of existing information on compounds such as SWCNT, MWCNT, fullerenes, silver, gold, titanium oxide, silicon oxide, and metallic mixtures in CNT. [504]. Furthermore, OECD published a review paper about the test guidelines and an overview of the materials tested, the test methods applied, and the discussions regarding the applicability of the OECD test guidelines, which are recognized methods for the regulatory testing of chemicals, including the compounds ZnO and nanoclays [507].

In the following period from 2017 to 2021, OECD focused on the environmental and consumer exposure to manufactured nanomaterials [508], exposure characterization [509], inhalation toxicity, human risk assessment, the biodurability of nanomaterials, and the different types of risk assessments (2018), establishing the physical-chemical parameters measurement, test guidelines [510] (2019) and biopersistence/biodurability of manufactured nanomaterials (2020) [511] and evaluating the tools and models used to assess environmental exposure besides the evaluation and categorization of the risk of nanomaterials functional assessment and statistical analysis (2021). In 2022. OECD deepened the sustainability and safe design [504]. Specifically, document 103 discusses topics of interest in the risk assessment of invoiced nanomaterials [512]. This document provides an overview of the chemical risk assessment paradigm and describes the adaptation of existing regulatory frameworks in various member countries. First, it summarizes information about the risk assessment of manufactured nanomaterials. It provides current practices, challenges, and strategies for the risk assessment of manufactured nanomaterials when data are limited. Finally, it concludes with the need for more research on specific risks, highlighting priorities for research toward assessing particular risks. Table 3 summarizes the main strategies, activities, and reports of OECD from 2006 to 2022, which are available online [504].

Despite the constant progress in the different lines of action of the OECD over the years, other issues are relevant, making it necessary to deepen the improvement of methods for detecting and characterizing complex matrices. The investigations were carried out thanks to the financing of the horizon 2020 program, reporting the findings made by different research groups [307,501,502,503,504,505,506,507,508,509,510,511,512,513], as discussed in the action cost CA 1714–2020 regarding “Protocols of consensus for the complete physicochemical characterization of new/existing chemical entities and/or nanomaterials” [514].

While establishing these strategies, OECD published other guidance documents on good practices of in vitro methods (GIVIMP). These documents aim to develop and implement in vitro methods for regulatory use in human safety assessment. Its beginnings go back to 2013, and the guiding document has been available since 2018 [515]. As the aspects highlight, the paper points out essential elements for knowing the nature of a certain nanomaterial, citing the appearance of the nanomaterial, nominal size, morphology, size distribution, aggregation, agglomeration phenomena, and surface characteristics (surface area, surface charge, surface chemistry). It also insists on evaluating the solvents’ concentration, the nanomaterial’s physicochemical properties and state, whether it is solid, liquid, or gas, the type of radiation, and all intermediate states, such as aerosol, dust, or viscous liquid. Other criteria include the type of preparation or the formulation of the tests before being applied in vitro. Other critical parameters must be considered, such as the composition, purity, pH, solubility, osmolality, lipophilicity, homogeneity, and photoreactivity. Furthermore, it includes recommendations about air handling, water supply, environmental control, heating, and cooling, always choosing to guarantee an adequate environment for the specific type of work in the laboratory [515]. Continued updates are available [516,517]. This documentation should be consulted within conservation activities when evaluating the effectiveness of a nanomaterial with biocidal purposes.

On the other hand, in 2015, the European Center for Ecotoxicology and Toxicology of Chemicals (ECETOC) Nano Task Force proposed a project entitled “*A Decision-making framework for the grouping and testing of nanomaterials*” (DF4nanoGrouping) [518]. The DF4nanoGrouping would cover all relevant aspects of a nanomaterial’s life cycle and biological pathways, i.e., intrinsic material and system-dependent properties, biopersistence, uptake and biodistribution, and cellular and apical toxic effects. The use (including the manufacture), release, and route of exposure are applied as “qualifiers” within the DF4nanoGrouping to determine if, e.g., nanomaterials cannot be released from a product matrix, which may justify the waiving of testing. The four main groups encompass (1) soluble nanomaterials, (2) biopersistent high-aspect-ratio nanomaterials, (3) passive nanomaterials, and (4) active nanomaterials. The DF4nanoGrouping aimed to group nanomaterials by their specific mode-of-action, which results in an apical toxic effect. This is eventually directed by a nanomaterial’s intrinsic properties [518]. The DF4nanoGrouping has been used in different applications, demonstrating a highly efficient means of identifying nanomaterials that may undergo risk assessment without further testing [519,520].

Furthermore, it is essential to highlight the role of private international standards for nanotechnology, such as the independent and non-governmental organization ISO (International Organization for Standardization). ISO established a technical committee for nanotechnologies in June 2005, structured into four working groups (WGs): WG 1: Terminology and nomenclature; WG 2: Measurement and characterization; WG 3: Health safety and the environment; and WG 4.

The Working Group on Health, Safety, and Environmental Aspects of Nanotechnologies (Working Group 3) defined the original structure. It served as the home of technical committee 229 for documentary standards related to nanotoxicology [488,503,521,522,523]. Among them, one of the main contributions was the ISO standard 29701:2010, “ISO 29701:2010”, which describes the application of a test using the Limulus amebocyte lysate (LAL) reagent for the evaluation of nanomaterials intended for cell-based in vitro biological test systems [524]. According to the norm, the test is suitable for use with nanomaterial samples dispersed in aqueous media, e.g., water, serum, or reaction medium, and for such media incubated with nanomaterials for an appropriate duration at 37 °C [524]. Although ISO standard 29701:2010 (upgraded in 2021) is restricted to test samples for in vitro systems, the methods can also be adapted to nanomaterials to be administered to animals by parenteral routes [524]. The ISO norm 29701:2010 stands out for its contributions to investigating and monitoring nanotechnology hazards. For example, advances using this standard made it possible to identify aspects of endotoxin contamination during the synthesis process and the handling of TiO_2_, silver, CaCO_3_, and SiO_2_ nanoparticles; all of these products are highly used in conservation processes. Specifically, the endotoxin extraction process—in this case, liposaccharides—was slowed down by the high concentration of nanoparticles since these nanomaterials can interfere with detection systems [525]. Other applications of the ISO norm 29701:2010 reported its use for controlling the toxicity of biomaterials by endotoxin contamination, which could be helpful, for instance, in bone regeneration using hydroxyapatite nanoparticles, a nanomaterial with several applications in cultural heritage [526].

### 7.1. Qualitative Evaluation Methods

Based on the concepts criteria established by the European Commission and OECD, the field of nanotechnology decided to apply its principles through the strategy known as control banding, based on the progress achieved with the qualitative or semi-quantitative tools previously developed for the pharmaceutical sector.

One of the goals of the control banding technique is to promote health and safety at work through a qualitative or semi-quantitative approach to risk assessment and management. It compiles schemes of existing data about the risks of different aggressive compounds, warning of the health harmfuls caused by their potential exposure. This technique provides a quick reference guide that, among its commitments, must be constantly updated. In this case, the term “banding” refers to bands of possible risks according to the degree of toxicity (high, medium, low) and exposures (small, medium, large exposure), which is very useful for companies according to their specific needs, depending on the type of compound, in the case of inhalation and contact with eyes or skin (skin/eye irritant, very toxic, carcinogenic, etc.) [527].

Along with the application of control banding techniques, different reports compile the continuous advances endorsed by NIOSH [528,529]. The best-known techniques are suitable for different groups, each fit for different purposes, different domain applications, and inclusion criteria [530]. Among them, the most used are the control banding Nano tool (United States), Stoffenmanager-Nano (The Netherlands), Nano safer (Denmark), Anses (France), the European Commission, and NEAT (NIOSH).

#### 7.1.1. Control Banding Nanotool

It is a qualitative risk assessment and management strategy associated with exposure to chemical products in the workplace. The concept consists of managing the potential exposures to harmful materials through the application of four control modalities. Furthermore, despite a large number of dangerous chemicals, only a limited number of prevention measures are available. Therefore, it is essential to consider the characteristics of the substance, the potential exposure, and the risk associated with the substance to determine the appropriate prevention strategy. If the potential harm to the worker increases, the prevention measures must be proportional to manage the risk [531]. Control banding is potentially valuable for risk management associated with nanomaterials in cases with no official occupational exposure limits [531].

The basis of this methodology is establishing the focus/receptor relationship according to the specific necessities [532,533,534,535]. For instance, this strategy indicates that the exposure depends on the air’s emission, transmission, and immission factors. Decisive exposure factors include tasks, local measures taken, general ventilation, and product characteristics, scored on a logarithmic scale.

The basis of this methodology is establishing the focus/receptor relationship according to the specific necessities. For this method, it is necessary to dispose of specific information on the nanomaterial that should be available in the safety data sheet and the product’s technical information. However, in the absence of this, the advice is to choose the less favorable option [536,537].

Even though it does not propose control measures associated with the risk (or priority) bands, the system allows the user to redesign the scenario to reduce the risk and test the risk reduction of the new preventive measure implemented. Examples of its application are the control of occupational exposure to nanoparticles in specific fields such as construction [538], chemistry laboratories [536,539], metallurgy, or industrial processes [533,540].

#### 7.1.2. Stoffenmanager Nano

The model uses information about physicochemical characteristics and mass balances to give a relative ranking of exposure situations. To guarantee a sound risk assessment process and the further acceptance of the Stoffenmanager, a comprehensive evaluation of its underlying exposure algorithm is highly desirable [541,542].

This strategy considers the source–receptor relationship [543] being adapted differently. The model emphasizes that the exposure depends on the air’s emission, transmission, and immission factors. Decisive exposure factors include tasks, local measures taken, general ventilation, and product characteristics, scored on a logarithmic scale.

This method considers that the specific information is available in the safety datasheets of the product and the technical information supplied with it. Therefore, if this is not the case, the method suggests choosing the most unfavorable situation [252].

This technique considers four domains: (1) the synthesis of nanomaterials; (2) powder handling; (3) spray and dispersions of ready-to-use nanoproducts; and (4) fracturing and abrasion of NM embedded in products. In addition, the user must assess the extent to which the substances are nano-relevant (defined as a particle size < 100 nm and products with a specific SA. ≥ 60 m^2^ g^−1^ [544].

Even though it does not propose control measures associated with the risk (or priority) bands, the system allows the user to redesign the scenario to reduce the risk and test the risk reduction of the new preventive measure implemented. Examples of its applications are the synthesis of nanomaterials such as TiO_2_ [536], alumina nanopowder [545], calcium carbonate [546], mortars elaboration [547], or the blast furnace process in steel-making plants [548].

The main European Commission’s objective is to give information that guarantees work safety with manufactured nanomaterials by using non-binding guidance for employers and health and safety professionals. It provides an overview of the problems of the safe use of intentionally manipulated nanomaterials in the workplace [549]. The European Commission collects a broad description of preventive action and provides a tool to comply with the specific aspects of worker prevention, such as risk assessment and management [198].

### 7.2. Semiquantitative Method: NEAT

The NEAT (Nanoparticle Emission Assessment Technique) is a method for measuring nanomaterials developed by NIOSH in 2009 [550] and later updated [551], which combines different techniques. It is semi-quantitative and conceptually designed to measure the concentrations of nanomaterials dispersed in the air in the workplace [552]. It considers the inhalation risk assessment in the production and handling of intentionally manipulated nanomaterials, considering the environment around the application. For instance, among its applications are the assessment of nanometal oxides emission [553] and the release of nanoparticles to the environment during the drilling of materials that include carbon nanotubes [554].

### 7.3. Dose Control

In addition to resolving issues related to the degree of the harmful of nanomaterials, it is necessary to assess the effects of the dose received.

The No Observed Adverse Effect Level (NOAEL) and the Low-Observed-Adverse-Effect-Level (LOAEL) approaches are traditionality applied to determine the point of departure (POD) of animal toxicology data for use in human health risk assessments. In this sense, NOAEL is suitable when the data are insufficient to support exposure–response modeling, as reported in acute inhalation exposures [555,556]. However, from the studies carried out by the United States Environmental Protection Agency (US EPA), it was possible to determine that its criteria are only sometimes valid—specifically, those related to the strict reliance on dose selection, the dose spacing, and the sample size used with critical effects [555,557]. For this reason, the US EPA decided to create a method that would estimate the benchmark reference dose (BMD), which, like the NOAEL, would serve as a starting point (POD) to derive the guideline value for human health. Nowadays, many health organizations around the world use BMD during their procedures. Particularly, a reference dose (BMD) is a dose or concentration that produces a predetermined change in the response rate of an adverse effect. This default change in the response is known as the baseline response (BMR) [558]. It was the moment when the Benchmark software emerged, which has evolved in its versions over the years [559]. This software allows for estimating the reference doses of nanomaterials [557]. BMD contributes to the reduction in experimental animals in toxicity studies [559]. However, some studies indicate that it has its restrictions since it depends on several parameters, including the data format presented or the consumed time [560]. Different organizations analyze the BMD [561,562,563], some of them comparing it with the traditional NOAEL, highlighting its advantages in case the data is unsuitable for BMD modeling [564]. However, certain studies indicate that there are also disagreements related to specific aspects of the modeling concerning the recommendations of the US EPA and the European Food Safety Authority (EFSA) [560].

One study applies the Benchmark dose to classify the sensitivity and toxicity of metal oxide nanoparticles in lung cells while providing information on the mode of action [558]. In this case, according to the BMD calculated for the most sensitive test, the toxicity decreased such that ZnO turned out to be less toxic, following an order of ZnO > CuO > TiO_2_ > ZrO_2_ > CeO_2_. The authors highlight that the BMD analysis was an effective tool for assessing the different aspects of risk [558]. However, as the authors maintain, there are still unresolved doubts. One of them is that the mechanisms of action of the different metallic nanoparticles are unknown in detail, nor are the ranges in which adverse effects occur, suggesting the necessity of carrying out in vitro toxicity tests [558]. Some specific in vitro and in vivo citometric studies apply the Benchmark dose analysis [565].

However, despite the progress, there is a need to continue deepening the development of BMD software, focusing on specific issues such as the implementation of new statistical methods in user-friendly software and the lack of consensus about how to derive the benchmark dose lower limit [566] Additionally, other associations insist on creating a BMD Standing Working Group [566,567]. Moreover, several reports speak of using BMD for information processing in several nanomaterials [568,569,570], such as ZnO or Siver [571], TiO_2_ [572], multiwalled carbon nanotubes [573], and SiO_2_ [574]. However, there is still a great variety of nanocomposites to be studied.

## 8. Recommendations for the Proper Handling and Storage of Nanomaterials: State of the Art

Another issue to resolve is how to prevent contact with nanomaterials. In this sense, it is necessary to take measures both for the workers handling the nanomaterials and to control the possible release of nanomaterials in the surroundings, including during the treatments using nanoproducts and the processes of synthesis, storage, or disposal of the same. In either case, its risk can affect humans, fauna, flora, or the environment close to the emission zone.

The control of contact by the respiratory route or the direct contact by the skin are the main aspects on which the standards focus.

The next section summarizes the different protection mechanisms that are currently commercially available. This compilation is the result of studies carried out by several research entities under the supervision of international organizations—mainly, by NIOSH and the European Commission, with the support of the different associations previously cited.

It is essential to bear in mind that the severity of the risk defines what type of protection is necessary, even if it is for occasional or frequent work. In all cases, the most crucial thing is to determine the dose to which the operator, people, animals, plants, and surrounding environment (soil, water) are exposed.

After reaching a consensus among the different organizations, the European Union developed several regulations that cover the control of personal protective equipment (PPE) and the installation of adequate filters to protect against the emission of nanomaterials in the workplace and external emissions to the environment. Details of this standard are available in the guide prepared by the European Commission in 2013 about protecting health, safety, and the potential risk of workers exposed to nanomaterials [198].

At the same time, NIOSH organized its guidance strategic plan for promoting the responsible development of nanotechnology [494]. In addition, various groups have published state-of-the-art articles over the years, commenting on the need to minimize risks and proposing new initiatives that leading organizations should consider [575].

Within the update reports, NIOSH 2022 established the following control measures: [493].

- Identify sources of potential ENM exposures
- Establish similar exposure groups by area or job tasks where workers may be exposed
- Characterize exposures of all potentially exposed workers
- Assess the effectiveness of engineering controls, work practices, personal protective Equipment (PPE), training, and other factors used to reduce or eliminate potential exposures.

The NIOSH report 2022 [406] established that the exposure monitoring should include these elements:

1. Develop an exposure assessment strategy.
2. Identify areas and tasks that are more likely to emit engineered nanomaterials, such as handling dry powders or the sonication of liquids. The use of direct reading instruments may assist with identifying these work areas.
3. Collect personal breathing zone (PBZ) samples for the worker’s full shift to determine adherence to the applicable REL.
4. Collect area samples using filter-based samples at indoor locations both in near proximity to and removed from the use of the engineered nanomaterials of interest to determine product migration and the extent of any cross-contamination (from production to non-production work areas) from work practices or improperly designed high vacuum or other ventilation systems.
5. Use task-specific short-term PBZ and area sampling to identify those tasks that are more likely to emit engineered nanomaterials.
6. Consult with the analytical laboratory to evaluate detection limits and sample time/volumes to achieve a sensitive enough measurement.

At the same time, the European Agency for Health and Hygiene at Work, through the Occupational Safety and Health Administration (OSHA), presents new updates for the management of nanomaterials in the workplace that are available through the Internet, among which the following should be highlighted [576]:

- Any situation in which nanomaterials may become airborne, such as the loading and unloading of nanomaterials or chemicals containing nanomaterials into/from milling or mixing equipment, the filling of chemicals into containers, the sampling of manufactured chemicals, and the opening of systems for product retrieval.
- The cleaning and maintenance of installations (including closed production systems) and of risk reduction equipment, such as filters in local exhaust ventilation systems.
- The research and development of nanomaterial-containing substances, such as composite materials.
- Handling powders and spraying mixtures containing nanomaterials. Powders are likely to have an increased risk of explosion, self-ignition, and electrostatic charging, giving rise to safety concerns. In addition, they may form dust clouds, leading to inhalation exposure.
- Mechanical or thermal treatment of items containing nanomaterials that could release because of these processes (e.g., laser treatment, grinding, or cutting).
- Waste treatment operations involving items containing nanomaterials.

A helpful guide recommended by the Environmental, Health, and Safety office of the Institute of Technology of the United States in 2009, based on NIOSH information, summarizes the best practices that, although in this case were established for the university sector, are equally valid for those groups that are unfamiliar with the subject [577]. It includes the following aspects:

- Prevent inhalation exposure
- Prevent dermal exposure
- Prevent laboratory contamination
- Prevent exposure during spills
- Obtain current toxicity information on nanomaterials in use.

The next section explains the most relevant aspects regarding personal protection equipment, ventilation control, nanomaterials isolation, specific regulations, and waste management.

### 8.1. Personal Protective Equipment (PPE)

One of the aspects that is essential to preventing damage from direct contact with nanomaterials is to have adequate personal protective equipment (PPE). This equipment must be used with frequency as part of prevention measures. In nanomaterials handling, its use results are mandatory when other preventive measures are insufficient to control exposure. Its use requires the application of a maintenance program and periodic reviews. The correct use of this equipment in security implies that users must know, in detail, the conditions of use, proper storage, and wear indicators that lead to their replacement [549].

The inhalation route is the main route of entry of nanomaterials into the body. As such, it requires a proportional effort when designing and implementing a Respiratory Protection Equipment management program (RPE).

In all senses, the objective of RPE is to reduce worker exposure to levels that are acceptable in the absence of adequate collective protections and during different circumstances. Therefore, it is essential to consider aspects such as the installation or maintenance of collective protections and short-term tasks that make collective impractical protections or emergencies. The risk situations associated with their conditions of use imply that the decision to use respiratory protection must consider different criteria. In this way, those provided by the professional judgment of an expert in occupational risk prevention, those that arise because of a risk assessment, and those that come from risk management practices are essential. The sum of the primary criteria intends to keep worker inhalation exposure below an internal control or exposure limit.

#### 8.1.1. Protective Clothing

The regulations of the European Commission, European Nanosafety [198], or the British [205] recommend using polyethylene textiles, preferably disposable ones, even if reusable, advising against the use of cotton, wool, or paper clothing, which can drop dust. Moreover, HSE states that this clothing type is the most convenient for workers in contact with CNTs and bio-persistent High-Aspect-Ratio Nanomaterials (HARNs) [578]. It is necessary to wear overalls that cover most of the body without exposing the skin during activities such as drilling, polishing, or cleaning due to the rise of the high content of particulate dust [198]. Furthermore, another aspect to consider is foot protection. In this case, as mentioned by the different standards, any classification of security work footwear could be valid as long as it provides the necessary level of tightness. In addition, it is necessary to use disposable footwear when the level of exposure requires it, which may also form part of the safety suit.

#### 8.1.2. Respiratory Protective Masks

Exposure to nanomaterials via the respiratory tract is a matter that requires particular interest. In this sense, it is crucial to consider that respiratory protection equipment is adequate when it can reduce the user’s exposure to an acceptable risk level [579]. During the years working with nanomaterials, various organizations and research groups have constantly been evaluating whether the quality of the masks is effective in controlling access to the organism [580,581,582]. Nowadays, it is possible to access the comparison of specific nomenclatures and selection criteria available for different international organizations [579,583,584,585,586]. There is even a new trend for creating nanostructured face masks for obtaining nanofibers that can incorporate multifunctional nanomaterials, which consist of polymeric nanofibers fabricated by electrospinning, phase separation, template synthesis, or self-assembly techniques, which include different nanoparticles such as metals (gold, silver, Zn), metal oxide (TiO_2_), metal composites (CuO-polyacrylonitrile), graphene, or carbon nanotubes [587]. For example, masks made by electro-spinnable polymers [588] include nanomaterials with antibacterial properties such as ZnO nanoparticles or titanium oxide nanowires, antiviral membranes containing Ag nanoparticles, and superhydrophobic masks including CNTs or graphene layers against several pathogens or organic compounds such as chitosan. In all cases, biodegradable, eco-friendly, and smart face masks are sought [588].

Nevertheless, this subject needs evaluation in the long term. Therefore, it is necessary to consider the possible reaction with the fibers that constitute it and the effectiveness of its adherence to avoid the eventual detachment and entry into the organism, besides the risk of dispersion to the environment in cases of the improper management of them.

The European Commission and NIOSH insist on the need for protection through high-efficiency facemasks, but their classification varies from efficiency to nanomaterial retention. In this case, the filters proposed by the European Union are those called FFP (Filtering FacePiece), which retain particles in the air, classifying them as FFP1 (Filters at least 80%), FFP2 (Filters at least 94%), and FFP3 (Filters at least 99%). Currently, the standard for protecting personnel at risk due to respiratory contact is the EN 149 standard of 2009 (an update to 2001) [589].

For its part, NIOSH classifies masks and respirators into three different alphanumeric types. In this way, depending on whether or not they are suitable for the presence of oil, there are N (not resistant to oil-based particles), R (resistant to oil-based particles), and P (oil-proof), with P being the most efficient. In addition, P masks resist up to 40 h or up to 30 days after the first time. Regarding the numbers, these refer to the percentage of the retention of nanoparticles, being 95 (retention up to 95%), 99 (retention up to 99%), and 100 (retention up to 99.97%).

There is also another type of nomenclature depending on each country. For example, in China, the GB 2626-2006 standard classification of non-oil-resistant masks is KN (95, 98, and 100) [498]. In Spain, the mask classification is P1 (low-efficiency filters), P2 (medium-efficiency filters), and P3 (high-efficiency filters) [579]. In the same way, the Australian and New Zealand regulations use the same classifications, P1, P2, and P3, with P3 being the most effective (AS/NZA 1716:2012) [590].

Currently, there are different designs of respiratory protection masks, self-filtering masks, disposable masks, half masks, full masks, external air-assisted, and self-contained masks, which can provide different levels of protection against airborne particles [591]. These levels of protection must be known a priori and protect against the entire range of environmental values the worker is subject to, be it daily, short-term, or ceiling exposure [494,591,592].

However, it is necessary to consider that filtration efficiency depends on specific parameters such as the particle size, charge, concentration, and flow rate through the filter material [593]. In addition, it is essential to note that contaminants can bypass the filter, passing through small gaps between the edge of the respirator and the face, known as “edge seal leakage”. The extent of leakage also depends on factors such as the size and shape of the face, facial hair, respirator design, and manner of use [593].

#### 8.1.3. Hand and Arm Protection

The skin is one of the main entry routes that is easily exposed when handling nanomaterials. Therefore, it is crucial to establish a barrier between the potentially harmful material and the skin. International associations recommend using polyethylene gloves because they are more resistant to the penetration of nanomaterials by diffusion than cotton or polyester gloves. In addition, latex, neoprene, or nitrile gloves resist the penetration of nanomaterials during an exposure time of only a few minutes [198,594]. However, it is essential to bear in mind that the effectiveness of gloves can vary depending on whether the particles are in powder or as colloidal solutions [595]. One example is the static and dynamic loading experiments using gloves with different compositions or thicknesses (nitrile, latex, neoprene, and butyl rubber) in powder form or colloidal solution [595]. The result showed how the particles in the colloidal solution penetrated through the nitrile gloves, which increased with the time of deformation, producing surface modifications due to the increase in the number, diameter, and depth of the pores [595]. In addition, the penetration of nanoparticles through gloves depends on the dissolution mediums. For example, the efficiency between nitrile rubber, butyl rubber, and latex gloves varies depending on the type of dissolvent (water, polyethylene glycol, as well as the glove thickness) [596]. Specific studies in nanoTiO_2_ dispersions comparing the efficiency of these gloves showed how the latex gloves had better efficiency for all types of solutions, similar to 200 µm-thick nitrile rubber gloves; it was very poor when they were 100 µm-thick [596]. Furthermore, in powder nanoparticles, the efficiency worsened in butyl rubber gloves and 100 µm nitrile rubber. The authors justify these differences because the chemical composition in this case was more favorable in latex gloves, and there was an increase in the protective barrier when the rubber nitrile gloves were thicker [596]. According to the researchers, there is a necessity to study different situations in the workplace, recommending a constant change, especially if the gloves are thinner or in cases of working with colloidal solutions [595]. Furthermore, NanoSafe recommends the use of nitrile for CNT, nitrile and neoprene for TiO_2_, and Pt, nitrile, neoprene, and vinyl for graphite [597].

The European Nano safe states that despite the degree of porosity of the different gloves, their efficiency at aerosols generated by nanomaterials is very high. Nano safe recommends establishing an appropriate selection of gloves based on their resistance to the nanomaterial and other chemical products or liquids with which the hands will come into contact [597]. As part of good practice, different organizations recommend changing the globes when they have visible signs of wear or contamination. Furthermore, their donning should ensure that no body parts are exposed.

Similarly, the correct way to manage the contaminated gloves is to store them in closed plastic bags in a designated isolated area until their removal as a waste. Moreover, it is essential to take care during the disposal of contaminated gloves to avoid skin contamination, as reported in several studies, including EEP clothing after handling nanomaterials.

The main recommendation is replacing disposable gloves frequently to reduce exposure to nanomaterials—for instance, when exposure to nanomaterials occurs in the liquid phase. In this case, the Health and Safety Executive [578] and the European Commission [198] consider single-use disposable gloves adequate. Furthermore, the glove material thickness is an important aspect to consider. One recommendation is to use at least two pairs of gloves when handling Carbon Nanotubes and High-Aspect-Ratio Biopersistent Nanomaterials [198].

#### 8.1.4. Eye Protection

Eye protection is essential but, at the same time, neglected, as some researchers point out [281]. The risk of entry through the eyes is very high and depends on the type of nanomaterial. Nowadays, the main recommendation is to work with universal frame glasses. The risk of splashing is greater during manipulation with colloidal solutions, which is why different associations recommend using face shields. When working with aerosols, it is best to use more full faces. Moreover, there are commercial glasses for protecting the eyes during the handling of nanomaterials.

All the associations recommend eye protection when handling any chemical product, including all nanomaterials. Safety glasses must have at least a closed integral frame, be well-fitting, and meet existing dust protection standards [198,205,494].

### 8.2. Laboratory Adaptation for Nanomaterials Processing and Storage

#### 8.2.1. Ventilation in Workplaces

One of the essential aspects to control is the emission of nanomaterials in the workplace. For this reason, it is necessary to maintain good ventilation and have an infrastructure capable of retaining the emission of particles to the outside. Thus, to obtain good ventilation, it is required to incorporate air filters into the workplace. The main objective of air filters is to eliminate particles. According to their configuration, mechanical and electrostatic filters have fibrous mediums or membranes to protect against other factors such as heating systems, air conditioning, and industrial applications. Its effectiveness depends on factors such as the fibers’ size, the filter’s density, or the material used. According to the results of different studies, it was possible to define four types of filter collection that catch the particles by diffusion, interception, inertial impact, and electrostatic attraction [598].

The diffusion mechanism bases its principle on random movements of the Brownian type so that the particle contacts the fiber through the filter. At interception, the radius of the particle moving along the line of the air stream is greater than the distance from the airline to the surface, which causes the particle to meet the fiber surface. When impact by inertia occurs, the air current curves around a fiber and the inertia of the particles make them continue to move straight so that they collide with the thread and adhere by an interchange of molecular forces [598].

Electrostatic attraction occurs when the particle and the fiber have opposite charges. In this case, there is a charge: mass dependency that is more effective if the particle size decreases. However, it is crucial to consider the effect of atmospheric conditions, which affect the capacity of nanoparticles to gain or lose electricity, and that the impact of atmospheric variability occurs faster than in microparticles [599], as observed in multiwalled carbon nano tubes and iron oxides dispersions [600].

It is essential to consider the filtration efficiency against the most penetrating particle size (MPPS). Theoretical efficiency approximates the movements of particles in the fibers of a filter. Thus, the actions of an aerosol around the vicinity of a filter depend on the particle size and fiber characteristics.

Moreover, experimental results indicate that the fraction of retained particles in the filter determines its efficiency. Therefore, it depends only on diffusion and inertia and not on the size of the holes in the filter. The fraction of particles retained in the filter determines its efficiency [598]. Nowadays, many studies promote the improvement of the air filter’s quality [601,602,603].

The European directive council of communities defined the workplace’s minimum health and safety standard [604]. The norms and other guidelines are available in subsequent compilations [605,606]. Following directive 89/654/CEE [604], all workplaces must comply with the minimum ventilation requirements, especially the ventilation of confined workplaces. The standard insists on guaranteeing sufficient fresh air in enclosed workplaces, considering the working methods used and the physical demands of the workers [604]. One of them is an adequate laboratory with extraction ventilation located directly in the manipulation place with nanomaterials. The specifications for this kind of ventilation must include the following conditions:

(1) Extraction cabin.
(2) Conduit that transports the contaminant along the extraction tube.
(3) Fan that moves air through the exhaust system.
(4) Smoke outlet where the system discharges the air.

The system must have filters capable of retaining particles. The standards for protection against the nanomaterials products also include the adequacy of fume cupboards, whose objective is to create a current of air inside the hood, with recirculation regions. There are several cupboards depending on the specific necessities [607,608,609,610,611]. If the main objective is the biological security [612], there are different configurations including outdoor evacuation or indoor recirculation considering the norm 12885, ISO TR, 2008 [613], the UK NanoSafety Partnership Group (UKNSPG), with contributions from the HSE [614].

The adaptation of insulating filters capable of retaining the particles emitted to avoid the external emission of nanomaterials in workplaces is an essential issue to consider.

The European Union standard EN-1822 classifies filters as EPA (Efficiency Particulate Air), HEPA (High-Efficiency Particulate Air), and ULPA (Ultra-Low-Penetration Air), EPA filters being the least efficient and ULPA filters being more efficient, with the lowest penetration, making them the most effective, along with, more recently, SULPA (Super Ultra-Low-Penetration Air) [615].

The term “filtration efficiency” considers the retention against the most penetrating particle size (MPPS). Theoretical efficiency approximates the movements of particles in the fibers of a filter. Thus, the actions of an aerosol around the vicinity of a filter depend on the particle size and fiber characteristics. However, experimental results indicate that the fraction of retained particles in the filter determines its efficiency. Therefore, it depends only on diffusion and inertia and not on the size of the holes in the filter [598]. Nowadays, many studies promote the improvement of the air filter’s quality [598,601].

The efficiency of each filter proposed by the EN 1822 standard [615] is the following:

EPA Filters:

E10 > 85% efficiency, <15% penetration (integral value)
E11 > 95% efficiency, <5% penetration (integral value)
E12 > 99.5% efficiency, 0.5% penetration (integral value)

HEPA filters:

HEPA 13 > 99.95% efficiency, <0.05% penetration (integral values); >99.75% efficiency, <0.25% penetration (local values)
HEPA 14 > 99.995% retention, <0.005% penetration ((integral values); 99.975% retention, <0.025% penetration (local values)

ULPA filters:

U15 > 99.995% efficiency, 0.0005% penetration (integral values); >99.975% efficiency, 0.0025% penetration (local values)
U16 > 99.9995% efficiency, 0.00005% penetration (integral value); >99.99975% efficiency, 0.00025% penetration (local values)
U17 > 99.99995% efficiency, 0.000005% penetration (integral value); >99.9999% efficiency, 0.0001% penetration (local values)

SULPA filter:

Super-Low-Penetrating Air filter with a minimum efficiency of 99.9999% on 0.12 µm particles (added later).

Following the recommendation of international agencies established in the European Norm EN 17141:2020 [616], currently, companies specialize in developing isolation or confinement equipment for handling nanomaterials in the workplace [617]. Thus, various equipment is available depending on the potential risk of environmental emissions that may affect the surrounding personnel. The fundamental thing is the adequacy of a cabin in which high-protection gloves are adapted for handling, avoiding contact with the nanomaterial, and keeping it isolated. Depending on their needs, each laboratory can use a pyramid portable glove bag for handling small amounts of nanomaterials which can be used in field works, glove box insulators for materials with high dispersion, or, if necessary, biological safety cabinets. In all cases, the most crucial thing is insulation, so depending on the risks, they can incorporate a fan to control any leakage and achieve tightness in the handling environment. In this way, any leak, such as the one that can occur due to a possible break in the gloves, can be controlled using inward constant gas flows to minimize the escape of the nanoparticles. Technical details and periodic reviews of the subject’s state are available in the literature, in which it is questioned or encouraged to improve its effectiveness [577,581,618].

#### 8.2.2. Organizational Measures in the Workplace: Labeling and Specifications

Controlling exposure to occupational risks is essential for protecting the workers and anyone who may be directly in contact with them.

Current regulations indicate that all nanomaterials must have a label including all possible information as chemical products in the work area. The storage must be in marked containers showing their chemical content and the most information. Likewise, storing nanomaterials that are dissolved in liquids or dry makes it mandatory to put them in unbreakable and completely sealed containers.

In addition, the accesses zone to nanomaterials handled and stored, including products or waste areas, must have indicative signs as pictograms, risks, obligations, prohibitions, information, and warnings information according to the applicable regulations.

#### 8.2.3. Nanoparticulate Waste Management

The management of nanomaterial waste is an aspect that requires high responsibility, taking into account the severe added damage that a wrong procedure can cause. There is a high possibility of generating large quantities of nano waste in the long term, which can easily affect living beings and ecosystems [619].

The current regulations [198] indicate the necessity to consider at least the following aspects:

1. Classify the waste within the families previously established or create a new one, taking into account the characteristics of the waste both for containing nanomaterials (solid, slurry, liquid) as well as by the composition of the dissolved medium (solvents, epoxies) and its shape.
2. A suitable container for the waste, which is required to be unbreakable, allows for an airtight seal; in eventual cases, the recommendation is to provide a second container according to the circumstances [198].
3. If the residue consists of easily dispersed dust in the air, it must adopt additional measures, such as the case of filling the container. This process must always be carried out within collective protection that acts on the focus and establishes a minimum time settlement of the dust generated inside the container. It can oscillate between half an hour and two hours, while the other option is to use a single-use container.
4. Label the container with the information associated with the risk of the collected waste.
5. Mark the container with a pictogram indicating the presence of nanomaterials and the risk associated with hazardous chemical agents.
6. Establish a temporary storage point enabled in this regard and comply with the table storage incompatibility established until its withdrawal by the authorized manager.
7. Establish the safety conditions and the mandatory PPE for handling and action in emergencies. For instance, in cases of cleaning spillages, there cannot be brushing, compressed air cleaning, or traditional vacuum cleaners aspirating in the workplace. In the last case, the recommendation is always to use vacuum cleaners including HEPA-filters [205].

## 9. Current Status of Regulations on the Protection of Cultural Heritage

The concern derived from the possible ecological impact of nanomaterials on cultural heritage has led some researchers to evaluate the current state of regulations regarding the leaching effect of nanomaterials in the environment [620]. Specifically, as discussed by Brunelli et al. (2021) in their review [620], further efforts are needed to assess the potential release of NPs by leaching to support an investigation of potential risks throughout their life cycle since the assessment. Nanotechnology-enabled materials and products’ safety and sustainability have not kept pace with their rapid commercialization [621].

The compilation by Brunelli et al. indicates that the registration, evaluation, and authorization of chemicals (REACH) regulation in force since 1 January 2020 (and the corresponding guidance manuals) includes a complete set of information on the physicochemical properties of MLs (e.g., distribution of particle size, shape, crystallinity, surface area, solubility/dissolution rate), as well as knowledge of environmental fate and toxicity. According to the REACH 2020 regulation, it is necessary to consider that twenty additional nano-specific information requirements must be met to register substances [622]. However, methods are needed to identify and investigate relevant exposure scenarios (including a justification for “no exposure” or “low exposure” assessments) in the event that a chemical safety assessment is required [620]. Previously, in 2018, the annexes to the European Union chemical legislation regarding nanomaterials were revised to provide more structure and clarity and oblige manufacturers, importers, and downstream users to make a considerable effort to understand the details of what should and should not be done [623]. As Clausen and Hansen (2018) point out, the annex revisions are very inclusive in some respects. In assessing persistence, bioaccumulation, and toxicity, registrants should consider all life cycle stages when making quantitative and qualitative estimates of the dose/concentration of the substance to which humans and the environment may be exposed. This revision includes estimating environmental distribution and fate and performing a characterization of the possible degradation, transformation, reaction processes, dissolution rate, particle aggregation and agglomeration, and changes in particle surface chemistry [623]. Among the decrees of the new annexes [623], it is worth highlighting the following issues:

1. Nanoform and characteristics that can influence (eco)toxicity and environmental exposure.
2. Do not solely use molecular structural similarities to justify grouping different nanoforms together.
3. Justify the relevance of the safety information provided for all registered nanoforms.
4. Document the safety of all registered nanoforms throughout the life cycle.
5. Provide information about test conditions and tested nanoforms.
6. Fulfill specific ecotoxicity-related test requirements for different nanoforms depending on their dissolution and solubility.
7. Comply with specific testing requirements related to toxicity for different nanoforms depending on their nature and likely route of exposure.
8. Consider multiple reporting metrics of results for nanoforms that are hazardous.
9. Justify waiving information requirements.
10. Propose additional testing and/or comply with ECHA testing requirements.

In addition, as highlighted by Brunelli (2021) [620], there are other aspects of the current regulations concerning the standard methods used to evaluate the leaching of NPs from nano-enabled products, such as ISO 2812:2018 [624], AWPA E11-97 (wood leaching) [625], and the work of the technical committee 351 CEN/TC through CEN/TS 16637-1:2018 [626], with which it is possible to identify adequate leaching tests for the release of harmful substances from construction products in soil, surface water, and groundwater.

## 10. Preventive Measures during Conservation Treatments of Cultural Heritage

**Figure 10:**
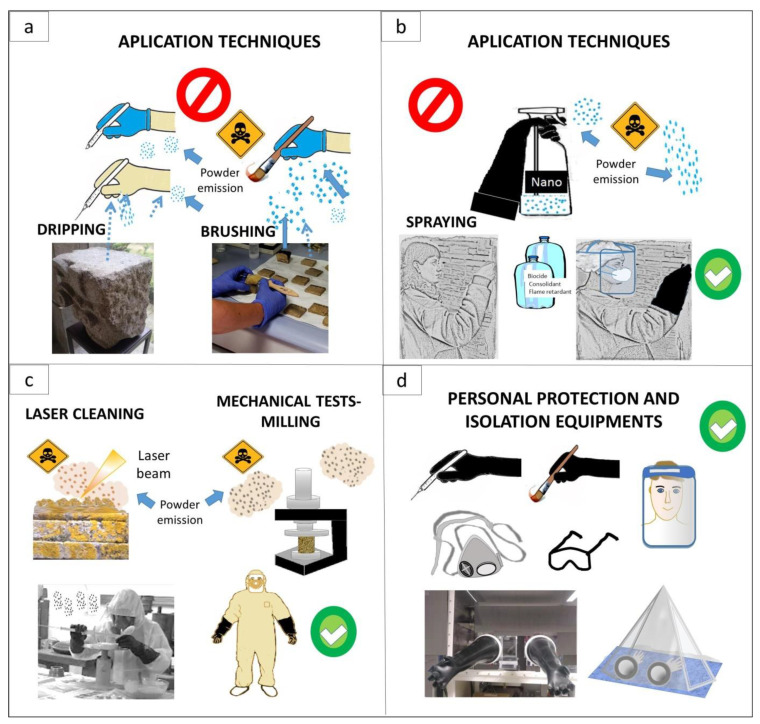
(**a**,**b**) Application methods of nanotechnology in cultural heritage conservation treatments and risks of toxicity. (**c**) Examples of powder emission of nanomaterials during laser cleaning, milling, or mechanical tests and adequate personal protection equipment recommendation. (**d**) Examples of Personal Protection Equipment (gloves, masks, face shield, protective glasses) and isolation measures (glove box, pyramid portable glove bag).

Based on the guidelines prepared by the different organizations, it is necessary to consider a series of precautions during the conservation procedures of heritage materials with nanomaterials. For example, actions that require procedures such as dripping, brushing, or spraying application methods of nanomaterials must consider that the operator is protected as much as possible from direct contact with the nanomaterial. Specifically, the risk of contact with the skin for nanoparticulate materials released into the environment when dripping or brushing treatments are used is more significant due to a wrong choice of protective gloves (Figure 10a), making it necessary to choose high-quality gloves. There is a high probability of inhalation during spraying treatments (Figure 10b). Moreover, there is a greater risk of nanomaterial emissions during cleaning processes—for example, by laser [128,131], mechanical tests, or milling procedures; the latter is commonly applied in breakdown nanomaterials synthesis processes [627]. Therefore, it is mandatory to take more significant protection measures due to the high risk of the emission of nanomaterials into the environment. It is necessary to use an approved protective suit that covers the entire body and feet, high-protection gloves, a hat that covers the head, and a mask and face shield (Figure 10c).

During dripping, brushing, and spraying procedures, it is necessary to protect the operator with a mask with an HEPA 14 filter, a face shield, gloves, and a protective suit (Figure 10d) [601]. In the case of procedures that do not require extensive nanomaterials manipulation or during optical visualization, to avoid contact with the eyes, it would be sufficient to use protective glasses. In all cases, it is best to have a glove box to handle nanomaterials and pieces subjected to nanomaterial treatments and to remain isolated to avoid contact with the outside. Furthermore, a pyramid portable glove bag could be convenient if it is necessary to transport nanomaterials, such as a work field or outdoor procedures. In addition, an adequate ventilation system and special containers for waste management must be available in the laboratory. Special care is necessary for handling the debris, based on the current international guideline recommendations, which have similarities to highly harmful chemical products [198,494].

It is essential to consider special care when cleaning brushes, paintbrushes, and any instruments, avoiding pouring their content into the water pipes; instead, they should be stored in labeled cans with information about their content and risk. Moreover, it is crucial to consider that the risk increases with the dose received, so in the case of frequent contact with nanomaterials, it is necessary to have a dosimeter for periodic control of the content of accumulated nanomaterials.

On the other hand, in any case, it is necessary to consider that the spraying, brushing, or immersion procedures are not generalized or standardized for a particular nano compound because each nanomaterial has its specific properties; a specific material might be applied with different methods depending upon a specific treatment [1,161,162,628]. Therefore, choosing the most appropriate method depends on its composition and textural characteristics, the water or environmental conditions, and the degree of deterioration. Other important parameters are the effectiveness of each method with regard to the consolidant penetration depth, mechanical properties, microstructure, contact angle, water sorptivity, color [628], number of applications, time interval between applications, and differences between the amount of the product absorbed and consumed, which depend on the degree of volatility of the solvents and the absorption capacity of the stone [162]. Thus, before starting the treatments, the main recommendation is to consider the possible risk of each procedure, contemplating the aerosol effect, including the evaporation of harmful organic solvents and release of nanoparticles during the spray coating process [629], the specific electric charge of nanoparticles and the meteorological variables [630,631,632]. Table 4 summarizes the main risks of toxicity depending on the application method and recommendations of security during the handling of products based on nanomaterials.

## 11. Final Considerations and Conclusions

After reviewing the state of the subject in terms of applications of nanotechnology in heritage conservation, it is necessary to consider some aspects that are relevant to confirm its degree of toxicity. This review article reports the main effects of nanomaterials frequently used in conservation treatments in their different fields without discarding many other signs of toxicity, which will be found as time goes by.

The main objective was to identify the possible toxicity risks during the handling, including brushing, spraying, or cleaning procedures. Another concern that is essential to consider is the release into the environment during other activities including crushing, grinding, and waste management. All of these processes are also applicable to different synthesis procedures or industrial activities.

It cannot be generalized that the toxicity of the analyzed nanomaterials is similar without taking into account their specific properties and functionalization, emphasizing a variation in their morphology, their aspect ratio, their roughness, or the size of nanomaterials. Although the nanomaterials have the same composition, they can cause different cytotoxic actions once their internalization occurs. Moreover, many researchers insist on the need for more specific physicochemical data, which have even given contradictory results due to the lack of detailed information on the nanomaterial. It is necessary to consider that when nanoparticles are taken into the body, each compound is in contact with varying dispersion media. Nowadays, there is a lot of knowledge about the solubility in water but less knowledge about organic solvents. Therefore, it is urgent to analyze the local environment through both in vivo and in vitro analysis, taking into account that a variation in the type of solvent can lead to changes in solubility and, therefore, more or less ionic propagation through the different cells.

There are gaps in other criteria, such as the analysis of the reactivity of nanomaterials, taking into account the degree of defects that ultimately make their difference.

The constant progress in the production of nanomaterials and hybrid nanostructured materials turns out to be subject to exploration in depth. It is mandatory to analyze criteria such as the incorporation of different compounds, which can lead to phase transformation processes that will vary their chemical and structural properties, symmetry, and unit cell size, conducing to variations in its efficiency, which in turn will affect the organism in a different way. Currently, there is not enough information on these specific properties.

Specifically, conservation work in the different lines frequently uses nanomaterials for consolidation processes, protection against aggressive biological agents, waterproofing, or fire retardants. Titanium dioxide and carbon nanotubes are highly toxic nanomaterials among the various compounds. Although several adverse effects are known for particular compositions, it is necessary to consider that, in new combinations, the degree of hazard is unknown. In this sense, it is also essential to consider that each nanomaterial behaves differently depending on the environmental conditions, both extrinsic and those produced when faced with different body fluids. Moreover, many nano compound mixes still need more studies, and part of this limitation depends on the detection limit of the characterization tools. On many occasions, there is limited access to specific body parts to track their behavior.

According to the stated reasons, it is crucial to consider the current work protection regulations during the handling and storage of nanomaterials. There should be no skimping on environmental protection measurements, as the long-term effects that nanomaterials can cause when released or transported through the environment is unknown.

Despite the fact that international organizations offer different guidelines for handling nanomaterials in different situations, it is necessary to constantly report the latest advances and update existing data to provide more information to users. In this sense, to achieve a more significant advance in the knowledge of nanotechnology and its toxicity, it is necessary to consider today’s contribution to diagnostic tools such as microscopic techniques. International reports need to expand their reporting, considering the constant support these techniques can offer.

Within international regulations, it is possible to consult the guides provided by different associations, such as the NIOSH, the European community, and the OECD, among others, for free. However, access to ISO standards from the private sector is limited.

On the other hand, after reviewing the state of the art concerning international or domestic regulations, there is an urgent need to carry out specific regulatory documents focused on conservation processes in which there are risks during spraying, brushing, and immersion procedures, as well as courses that require cutting, polishing or cleaning the surface, such as laser treatments, and other in which there is a high probability of emission of nanoparticles into the environment. In this sense, it is urgent to establish a committee of experts in nanotechnology applied to heritage, with knowledge of toxicity and nano prevention, focused on establishing standards and practical guides and defining the specific dose control of each nanomaterial for the different research fields.

Although nanotechnology offers other advantages in terms of protection, such as the latest advances in personal protective equipment with nanomaterials, it is urgent to assess its compaction and the eventual airborne release, as well as the management of the waste of this new emerging technology. The same occurs with the adaptation of sensors with nanomaterials for monitoring gases in closed environments.

One of the emerging problems is the storage of waste from nanomaterials. Although international organizations report some guidelines, it is urgent to exercise a collective plan to delve further into the toxicity issue since its reactivity continues to be high as time passes. Therefore, it is crucial to consider that the time elapsed since the work with nanomaterials began differently from what will come in the future. Consequently, it is essential to decide what to do with these wastes, since when different compounds are in contact, there will be chemical reactions between them, and it is unknown to what point their toxicity may increase. In addition, it is necessary to deepen the different alternatives regarding the management of nanomaterial waste so as not to cause damage to the water cycle, farmland, and living beings, since, in this way, the damage would further increase globally.

## Figures and Tables

**Table 3:** Main strategies, activities, and reports of OECD from 2006 to 2022.

Year/ Period	Strategies, Activities	Report No.
2006–2015	Definition of criteria for the safety of manufactured Nms Design of testing guidelines on: The ecotoxicology and environmental fate of manufactured Nms Inhalation toxicity tests Genotoxicity Preliminary guidance about advances in the safety of manufactured Nms	1 [505] 62 [506]
2016	Categorization of nanostructured materials, physical-chemical parameters, methods for regulating nanomaterials SWCNT/MWCNT fullerenes, silver, gold, titanium oxide, silicon oxide, and metallic compounds in CNT	63–79 [504]
2017	Sampling strategies, techniques, and protocols for determining the concentrations of manufactured nanomaterials in the workplace air	80–84 [504]
2018	Inhalation toxicity of submicron particles, in vitro methods for human hazard assessment, biodurability of nanomaterials, and different types of risk assessments of manufactured nanomaterials	85–88 Test Guidelines 318, 412–413 [504]
2019	Physical-chemical parameters measurement	89–91 [504]
2020	Biopersistence/biodurability of manufactured nanomaterials, categorization of Nms risks	92–97 [511]
2021	Evaluating tools and models used to assess the environmental exposure to manufactured Nms Functional evaluation and statistical analysis	98–102 [504]
2022	Sustainability and safe design	103–105 [512]

**Table 4:** Main risks of toxicity depending on the application method and safety recommendations during the handling of products based on nanomaterials.

Procedure	Risk	Reccomendations
Spraying	Dispersion through the air of nanoparticles: contact with the skin, inhalation. Evaporation of harmful organic solvents and release of nanoparticles [629]	Avoid the applications of powders of nanoparticles [198] Individual protection equipment: gloves, mask (HEPA 14), and special clothing [579] Avoid the applications of powders of nanoparticles [198] Use specific containers for waste management [580,581,582] Adapt the area with isolation equipment and ventilation control indoors and outdoors [631]
Brushing	Skin exposure to nanoparticles	
Inmersion	Splash and dispersion though the air, soils, and rivers, contact with the skin, inhalation [257,630]	
Cleaning, milling	Dispersion through the air of nanoparticles: dermal and ocular contact, inhalation [631]	

## Data Availability

The information presented in this review is a compendium of the existing literature about the research of different groups and associations in Cultural Heritage conservation, nanotoxicity and preventive measures, gaps, or unresolved problems up to date.
